# FGF2 supports NANOG expression via pyruvate dehydrogenase–dependent histone acetylation under low oxygen conditions

**DOI:** 10.3389/fcell.2025.1623814

**Published:** 2025-10-28

**Authors:** Petr Fojtík, Martin Senfluk, Katerina Holomkova, Anton Salykin, Jana Gregorova, Pavel Smak, Ondrej Pes, Jan Raska, Deborah Beckerova, Monika Stetkova, Petr Skladal, Miroslava Sedlackova, Ales Hampl, Dasa Bohaciakova, Stjepan Uldrijan, Vladimir Rotrekl

**Affiliations:** ^1^ Department of Biology, Faculty of Medicine, Masaryk University, Brno, Czechia; ^2^ International Clinical Research Center (ICRC), St. Anne’s University Hospital, Brno, Czechia; ^3^ Department of Histology and Embryology, Faculty of Medicine, Masaryk University, Brno, Czechia; ^4^ Department of Biochemistry, Faculty of Medicine, Masaryk University, Brno, Czechia; ^5^ Department of Biochemistry, Faculty of Science, Masaryk University, Brno, Czechia

**Keywords:** pyruvate dehydrogenase - PDH, histone acetylation, nanog, FGF2, pluripotency, ROS - reactive oxygen species

## Abstract

**Introduction:**

The safe and effective application of human pluripotent stem cells (hPSCs) in research and regenerative medicine requires precise control over pluripotency and cell fate. Pluripotency is characterized by high histone acetylation and aerobic glycolysis, while differentiation involves metabolic remodeling and reduced acetylation. Pyruvate dehydrogenase (PDH) links these processes by converting glycolytic pyruvate into acetyl coenzyme A (Ac-CoA), the key substrate for histone acetylation.

**Methods:**

We investigated how PDH activity regulates histone acetylation and pluripotency maintenance under physiologically relevant oxygen levels (5% and 21% O₂). PDH contribution to histone acetylation was assessed using a specific PDH inhibitor, followed by rescue experiments with acetyl-CoA precursors. hPSCs were exposed to variations in FGF2 signaling and reactive oxygen species (ROS) using H₂O₂ treatment to evaluate redox-dependent modulation of PDH and downstream effects on pluripotency factors. Protein levels and post-translational modifications were analyzed by Western blotting and quantitative PCR, relative metabolite concentrations by LC–MS, and ROS levels by fluorescence microscopy.

**Results:**

Active PDH promoted global histone H3 acetylation and upregulated the expression of the pluripotency factor NANOG, specifically under 5% O₂. Mechanistic analysis revealed a novel FGF2–MEK1/2–ERK1/2–ROS signaling axis that regulates PDH activity through redox-sensitive mechanisms. This regulatory pathway was oxygen-dependent and absent under atmospheric oxygen levels (21% O₂).

**Discussion:**

These findings identify PDH as a redox-sensitive metabolic switch connecting cellular metabolism with the epigenetic control of pluripotency by modulating Ac-CoA availability.

**Conclusion:**

Our study highlights the importance of oxygen tension, ROS homeostasis, and growth factor signaling in shaping the metabolic–epigenetic landscape of hPSCs, with implications for optimizing stem cell culture and differentiation protocols.

## 1 Introduction

Human pluripotent stem cells (hPSCs) are invaluable tools for developmental research, drug testing, and potential regenerative medicine due to their ability to self-renew indefinitely and differentiate into all cell types of the human body. However, leveraging these properties safely and effectively depends on precisely controlling their fate determination, which remains challenging due to their heterogeneity even among morphologically indistinguishable populations (Hayashi et al., 2019; Kalmar et al., 2009; Nguyen et al., 2018). Much of this heterogeneity stems from differences in epigenetic modifications (Carter and Zhao, 2020). Chromatin-opening histone modifications, notably histone acetylation, are upregulated in hPSCs to maintain their plasticity and are considered hallmarks of pluripotency (Huang et al., 2016; Mu et al., 2015; Trisciuoglio et al., 2018). hPSCs heterogeneity can also be observed as a fluctuation of pluripotency factors, mostly reported for NANOG (Hayashi et al., 2019; Kalmar et al., 2009; M. Li and Izpisua Belmonte, 2018). However, pluripotency is also regulated by factors such as energy metabolism (Tsogtbaatar et al., 2020), cytokine signaling (Dvorak et al., 2005), oxygen tension (Forristal et al., 2010), and reactive oxygen species (ROS) (Fojtík et al., 2021), which are intricately interconnected with histone acetylation.

Histone acetylation relies on the availability of acetyl coenzyme A (Ac-CoA), a substrate for histone acetyltransferases (Jo et al., 2020). In hPSCs, glycolysis is the primary source of Ac-CoA for histone acetylation (Moussaieff et al., 2015). Pyruvate, the end product of glycolysis, is converted into Ac-CoA in mitochondria by the pyruvate dehydrogenase (PDH), which can either fuel the tricarboxylic acid (TCA) cycle and oxidative phosphorylation (OxPhos) or be transported to the cytosol and nucleus via a citrate shuttle to take part in lipid synthesis or enable histone acetylation. PDH has also been found in the nucleus, where it directly supplies Ac-CoA for histone acetylation, and interestingly, the regulation of mitochondrial and nuclear PDH differs (Nagaraj et al., 2017; Sutendra et al., 2014).

Unlike most somatic cells, hPSCs preferentially utilize glycolysis over OxPhos for energy production even in 21% O_2_ (Tsogtbaatar et al., 2020; Varum et al., 2011), allowing the excess Ac-CoA to potentially support histone acetylation. The PDH acts as a key metabolic switch between glycolysis and OxPhos, with its activity regulated via phosphorylation by pyruvate dehydrogenase kinases (PDHKs) and dephosphorylation by pyruvate dehydrogenase phosphatases (PDPs) (Korotchkina and Patel, 2001; Patel and Korotchkina, 2006). Inhibition of PDHKs or activation of PDPs has been shown to promote histone acetylation in other models (Karagiota et al., 2022), suggesting that PDH regulation could be critical for managing histone acetylation in hPSCs (Yucel et al., 2019).

The maintenance of pluripotency *in vitro* depends on cytokine-triggered signaling cascades, including fibroblast growth factor 2 (FGF2), which activates pluripotency genes through MEK1/2-ERK1/2 and PI3K/AKT pathways (Geary and Labonne, 2018; Haghighi et al., 2018; J. Li et al., 2007; Wang et al., 2017). These pathways not only regulate key transcription factors such as OCT-4, SOX2, and NANOG (Kelly et al., 2020) but also enhance glycolytic metabolism in hPSCs. ERK1/2 regulates key glycolytic enzymes (Papa et al., 2019; Yang et al., 2012), while PI3K/AKT signaling, together with mTOR, supports glucose uptake, nutrient sensing, and anabolic processes, further promoting glycolytic flux (Gottlob et al., 2001; Robey and Hay, 2009; Saxton and Sabatini, 2017; Zhou et al., 2020). Therefore, by promoting glycolysis, FGF2 can support Ac-CoA production, linking energy metabolism with pluripotency maintenance.

Oxygen tension also influences pluripotency and metabolic preferences. Reduced oxygen levels (∼5% O_2_), similar to early embryonic physiological conditions (Fischer and Bavister, 1993; Sciorio and Smith, 2019), promote hPSC maintenance, prevent differentiation, and even reverse differentiation when applied to committed cells (Fojtík et al., 2021; Lin et al., 2006; Mathieu et al., 2013; Närvä et al., 2013; Yoshida et al., 2009). Furthermore, low oxygen levels enhance glycolytic metabolism over OxPhos in hPSCs (J. Kim et al., 2006). This shift is mediated by hypoxia-inducible factors (HIFs), which upregulate PDHKs leading to inhibition of PDH, suppression of TCA cycle activity, and reduced OxPhos. At the same time, HIFs promote the expression of glycolytic enzymes (J. Kim et al., 2006; Semenza, 2001; Semenza et al., 1996). Moreover, reduced oxygen tension modulates FGF2 signaling by lowering ROS levels (Fojtík et al., 2021), linking oxygen availability with FGF2-dependent regulation of energy metabolism and production of Ac-CoA for histone acetylation.

ROS further contribute to this regulatory network. While high ROS levels can be damaging, physiological ROS levels act as signaling molecules (Sies and Jones, 2020; Sinenko et al., 2021) modulating pathways such as MAPK and PI3K/AKT (Fojtík et al., 2021; J. H. Kim et al., 2018; Kučera et al., 2017; Okoh et al., 2013), influencing glycolysis (Mullarky and Cantley, 2015; Samanta and Semenza, 2017), PDH activity (Cesi et al., 2017), and pluripotency maintenance (Fojtík et al., 2021; Guo et al., 2004; Shen et al., 2024; Zhang et al., 2016).

### 1.1 Hypotheses and objectives

Pluripotency, histone acetylation, cellular metabolism, oxygen levels, ROS, and FGF2 signaling are intricately connected within a complex regulatory network. In this study, we aimed to elucidate the role of PDH in producing Ac-CoA for histone acetylation, and to assess its downstream impact on pluripotency maintenance by focusing on the expression of NANOG, a core pluripotency marker known for its dynamic fluctuation in hPSC cultures (Hayashi et al., 2019; Kalmar et al., 2009; M. Li and Izpisua Belmonte, 2018) with particular sensitivity to histone acetylation (Hattori et al., 2007; Horne et al., 2014; M. S. Kim et al., 2015).

Given that glycolysis is the primary source of Ac-CoA for histone acetylation in hPSCs (Moussaieff et al., 2015), we hypothesized that modulating PDH activity would significantly affect both histone acetylation and NANOG expression. Additionally, we investigated how PDH is regulated by factors associated with pluripotency, including FGF2 signaling and oxygen availability (Eiselleova et al., 2009; Haghighi et al., 2018; Mathieu et al., 2013; Närvä et al., 2013), both of which are known to influence glycolytic metabolism (Fumarola et al., 2017; Liu et al., 2018; Papandreou et al., 2006).

Finally, building on our previous findings that ROS modulate FGF2 signaling and suppress pluripotency in hPSCs (Fojtík et al., 2021), we further explored whether ROS levels also regulate PDH activity and histone acetylation, aiming to unravel the complex interplay between metabolic regulation, redox signaling, and epigenetic control in maintaining hPSC pluripotency.

## 2 Methods

### 2.1 Cell culture

Experiments were performed using the human embryonic stem cell (hESC) lines CCTL12 (RRID:CVCL_C858) and CCTL14 (RRID:CVCL_C860), and the human induced pluripotent stem cell (hiPSC) line AM13, previously characterized (Adewumi et al., 2007; Krutá et al., 2014). Since we used both hESCs and hiPSCs in our study, we are using and overarching human pluripotent stem cells (hPSCs) term in this article.

For long-term maintenance, hPSCs were cultured on mitotically inactivated mouse embryonic fibroblasts (MEFs) derived from CD1 or CF1 mouse strains, using human embryonic stem cell medium (hESCM). The hESCM consisted of DMEM/F12 (Thermo Fisher Scientific, 21,331–020), supplemented with: 15% (v/v) knockout serum replacement (Thermo Fisher Scientific, 10,828–028), Non-essential amino acids (Thermo Fisher Scientific, 11,140–035), L-glutamine (0.5% v/v; Biosera, XC-T1715), Penicillin-streptomycin (Biosera, XC-A4122), 2-mercaptoethanol (Sigma-Aldrich, M3148), and FGF2 (4 ng/mL; PeproTech, 100-18B). Cells were cultured in a colony-type format under standard conditions.

For experimental treatments, hPSCs were transitioned to a feeder-free monolayer culture on Matrigel hESC-qualified Matrix (Corning, 354,277)-coated dishes and maintained in MEF-conditioned hESCM medium (CM+) supplemented with FGF2 (10 ng/mL).

CM+ was prepared by incubating hESCM on mitotically inactivated MEFs for 24 h. The same MEF-containing dish was reused for seven consecutive days; media from all seven batches were then pooled, supplemented with L-glutamine (0.5% v/v), and filtered. A parallel MEF-conditioned medium without FGF2 (CM-) was prepared identically, but using hESCM lacking FGF2 and without further supplementation.

Cells cultured on Matrigel were maintained for a maximum of seven passages. For hypoxic conditions, cells were cultured at 5% O_2_ in a MCO-18M multigas incubator (Sanyo, Japan). Passaging methods depended on the culture system. On MEFs, cells were passaged mechanically as clumps. On Matrigel, cells were dissociated using TrypLE Express Enzyme (Thermo Fisher Scientific, 12605010) 2 min/37 °C, dissociated in fresh media, centrifuged at 200 *g* for 4 min at 4 °C, and resuspended in fresh media.

A list of all compounds used for experimental treatments, including working concentrations, is provided in Table 1.

**TABLE 1:** List of compounds used to treat the hPSCs in this study.

Name	Abbreviation	Work concentration	Effect	Cat. #	Manufacturer
PD0325901	PD03	0.2 µM	MEK1/2 inhibition	PZ0162	Sigma-Aldrich
SC79	SC79	10 µM	AKT activation	S7863	Selleck Chemicals
CPI-613	CPI	10 µM	PDH inhibition	S2776	Selleck Chemicals
Hydrogen peroxide	H_2_O_2_	5 µM	ROS induction	H1009	Sigma-Aldrich
Glutathione (reduced)	GSH	5 mM	ROS quenching	G6013	Sigma-Aldrich
Sodium citrate	Cit	5 mM	Ac-CoA supplementation	W302600	Sigma-Aldrich
Sodium acetate	NaAc	5 mM	Ac-CoA supplementation	935,700	Sigma-Aldrich

### 2.2 Gene silencing

Endoribonuclease-prepared small interfering RNAs (esiRNAs) targeting *PDP1* (10 nmol; Sigma-Aldrich, NM_018444: SASI_Hs01_00128068) were transfected into hPSCs using Lipofectamine 2000 (Thermo Fisher Scientific, 11,668–037), following the manufacturer’s instructions.

Successful knockdown was confirmed by Western blot analysis approximately 48 h post-transfection, and 2 h after media change.

### 2.3 Western blotting

hPSCs cultured on Matrigel-coated dishes were harvested at a maximum of 70% confluence. Cells were washed three times with 1× phosphate-buffered saline (PBS) and lysed on ice using 1% SDS lysis buffer (50 mM Tris-HCl, 1% SDS, pH 6.8). Protein concentrations were determined using the DC Protein Assay (Bio-Rad, 5000111) and measured in triplicates using a DTX 880 Multimode Detector (Beckman Coulter).

Protein concentrations were normalized to 1 mg/mL, followed by the addition of 10× Laemmli sample buffer. Lysates were briefly boiled and resolved by SDS-PAGE using either 8% or 10% polyacrylamide gels, with 15 µg of total protein loaded per lane. Electrophoresis was performed at 140 V for 70 min. Proteins were transferred to Immobilon-P PVDF membranes (Merck Millipore, IPVH00010) using a wet transfer system at 100 V for 60 min.

Membranes were blocked in 5% non-fat dry milk prepared in TBS-T (Tris-buffered saline with 0.1% Tween-20) for 1 h at room temperature, then incubated overnight at 4 °C with primary antibodies diluted in the same blocking solution. The next day, membranes were washed three times for 15 min in TBS-T and incubated with secondary antibodies, also diluted in blocking buffer, for 1 h at room temperature, followed by five washes of 10 min each in TBS-T.

Detection was performed using Immobilon Western Chemiluminescent HRP Substrate (Merck Millipore, P90720). Images were acquired using the G:Box Chemi imaging system (SYNGENE, Bangalore, India). Image adjustment was done using GIMP2 software, and densitometric analysis was carried out using ImageJ.

For analysis of PDP1 oxidation-induced mobility shifts, cells were lysed in native lysis buffer (100 mM Tris, pH 7.0; 150 mM NaCl; 1 mM EDTA; 0.1% Triton X-100), supplemented with cOmplete Mini Protease Inhibitor Cocktail (Roche, 11836153001) and 50 mM N-ethylmaleimide (Sigma-Aldrich, 04,259). Samples were sonicated, mixed with non-reducing loading buffer, and resolved on 8% SDS-PAGE at 4 °C. Protein transfer and detection were carried out as described above.

A complete list of primary and secondary antibodies and their dilutions used in this study is provided in Table 2. Western blot quantification was performed using ImageJ, as previously described (Fojtík et al., 2021), and target proteins were normalized to appropriate loading controls.

**TABLE 2:** List of primary and secondary antibodies used in Western blotting.

Primary antibodies				
Target	Dilution	RRID	Cat. #	Manufacturer
ERK1/2	1:1000	RRID:AB_330744	9102	Cell Signaling Technology
pERK1/2	1:1000	RRID:AB_331646	9101	Cell Signaling Technology
AKT	1:1000	RRID:AB_329827	9272	Cell Signaling Technology
pAKT	1:1000	RRID:AB_329825	9271	Cell Signaling Technology
α-Tubulin	1:2000	RRID:AB_10734943	11–250-C100	Exbio
PCNA	1:2000	RRID:AB_10602096	HPA030522	Sigma-Aldrich
pan-AcH3	1:2000	RRID:AB_873860	47,915	Abcam
H3	1:1000	RRID:AB_331563	9715	Cell Signaling Technology
AcH3K9	1:1000	RRID:AB_3085344	29133-1-AP	Proteintech
AcH3K27	1:1000	RRID:AB_3670631	82902-1-RR	Proteintech
PDH	1:500	RRID:AB_2162928	2784	Cell Signaling Technology
pPDH (Ser^293^)	1:1000	RRID:AB_10616069	AP1062	Calbiochem
PDHK1	1:1000	RRID:AB_1904078	3820	Cell Signaling Technology
PDP1	1:1000	RRID:AB_2799686	65,575	Cell Signaling Technology
Vinculin	1:2000	RRID:AB_2728768	13,901	Cell Signaling Technology
Nanog	1:500	RRID:AB_2150401	Sc-33759	Santa Cruz Biotechnology
Secondary antibodies				
Name	Dilution	**RRID**	Cat. #	Manufacturer
anti-rabbit IgG-HRP	1:3000	RRID:AB_2099233	7074	Cell Signaling Technology
anti-mouse IgG-HRP	1:5000	RRID:AB_390192	12–349	Merck Millipore

### 2.4 Analysis of reactive oxygen species (ROS) levels using CellROX green

CellROX Green is a fluorogenic reagent for detection and quantification of ROS. Unlike comparable dyes (e.g., H_2_DCFDA) it is aldehyde-fixable, can be added to a complete media, and is resistant to detergents. While CellROX Green does not distinguish between specific ROS types, it provides a general measure of overall oxidative stress by detecting the products of various ROS reactions with the dye.

hPSCs grown on Matrigel-coated glass coverslips were deprived of FGF2 for 24 h prior to experimental treatment. Fifty minutes before the end of experimental treatment, CellROX Green reagent (5 μM; Thermo Fisher Scientific, C10444) was added directly to the culture medium. After incubation, cells were washed three times with ice-cold PBS and fixed with 4% paraformaldehyde (Sigma-Aldrich, 158,127) for 30 min at room temperature in the dark.

Fluorescence imaging was performed within 6 h of fixation using a Zeiss LSM700 confocal microscope equipped with a ×40 1.3 oil DIC objective lens (Carl Zeiss, Oberkochen, Germany). Snapshots were taken under identical acquisition settings, including exposure time and gain, to ensure consistency across all samples. Images were acquired from approximately equal-sized cell clusters.

Data were collected from five independent biological replicates, each consisting of 10–15 images per condition, with equal image counts across all conditions within each experiment. ROS levels were quantified as total fluorescence intensity divided by fluorescence area in raw images using ImageJ. Resulting values were normalized to the control average within each replicate and then pooled for final plotting and analysis.

### 2.5 Analysis of reduced GSH levels using monochlorobimane

Monochlorobimane (mBCl; Sigma-Aldrich, 69899) is a thiol-reactive fluorescent probe that selectively binds to reduced glutathione (GSH), forming a fluorescent adduct suitable for flow cytometric analysis (Hedley et al., 1990). As such, it provides a complementary approach for assessing the cellular redox state. hPSCs cultured on Matrigel-coated plates were incubated with 100 µM mBCl for 30 min at 37 °C in complete media, followed by dissociation using TrypLE Express. Cells were resuspended in ice-cold PBS and immediately analyzed using a Beckman Coulter Cytomics FC 500 flow cytometer (Beckman Coulter, Brea, CA, USA). Singlet cells were gated based on forward and side scatter properties, and a minimum of 10,000 events was recorded per sample. Fluorescence intensity was measured on a logarithmic scale, and the median fluorescence intensity was used for quantification. Data were analyzed using FlowJo software (version 7.2.2; FlowJo, Ashland, OR, USA) and relativized to the control condition (FGF2-treated cells).

### 2.6 RNA isolation and quantitative real-time PCR (qRT-PCR)

Total RNA was extracted using RNA Blue reagent (Top-Bio, Czech Republic) according to the manufacturer’s instructions. RNA concentration and purity were assessed using a NanoDrop spectrophotometer (NanoDrop Technologies, Wilmington, Germany).

For cDNA synthesis, 2 μg of total RNA were reverse-transcribed using Moloney Murine Leukemia Virus (M-MLV) reverse transcriptase (Invitrogen, Carlsbad, CA, USA) and Oligo (dT) primers (Thermo Fisher Scientific, USA) at 37 °C for 1 h, followed by enzyme inactivation at 85 °C for 5 min.

Quantitative real-time PCR was performed using the LightCycler® 480 DNA SYBR Green I Master Mix (Roche) on a LightCycler 480 instrument. Gene expression data were normalized to GAPDH mRNA levels and calculated using the 2^−ΔCq^ method.

Primer sequences used for qRT-PCR are listed in Table 3.

**TABLE 3:** List of primers used in qRT-PCR.

Gene		Sequence
*GAPDH*	forward	5′-AGCCACATCGCTCAGACACC-3′
	reverse	5′-GTACTCAGCGCCAGCATCG-3′
*NANOG*	forward	5′-CCTATGCCTGTGATTTGTGG-3′
	reverse	5′-CTGGGACCTTGTCTTCCTTT-3′
*SOX2*	forward	5′-TACAGCATGTCCTACTCGCAG-3′
	reverse	5′-GAGGAAGAGGTAACCACAGGG-3′
*POU5F1*	forward	5′-CTGGGTTGATCCTCGGACCT-3′
	reverse	5′-CCATCGGAGTTGCTCTCCA-3′

### 2.7 Mitochondrial membrane potential measurement

Mitochondrial membrane potential was assessed using the fluorescent probe tetramethylrhodamine methyl ester (TMRM) (Invitrogen, T668). TMRM is a cationic, membrane-permeable dye that accumulates in the mitochondrial intermembrane space in proportion to the proton gradient, and its fluorescence intensity reflects the mitochondrial membrane potential.

TMRM was added directly to the culture medium at a final concentration of 20 nM and incubated with cells for 20 min at 37 °C. Cells were then washed with 1× PBS and harvested using TrypLE Express. After centrifugation, the supernatant was discarded, and the cell pellet was resuspended in 0.5 mL ice-cold PBS. All subsequent steps were performed on ice.

Samples were analyzed immediately using a Beckman Coulter Cytomics FC 500 flow cytometer (Beckman Coulter, Brea, CA, USA). Cell singlets were gated based on forward and side scatter properties, and at least 10,000 events were recorded per sample. Fluorescence was measured on a logarithmic scale, and median fluorescence intensity was used for quantification. Data were analyzed using FlowJo software version 7.2.2 (FlowJo, Ashland, OR, USA).

### 2.8 Mitotracker Red CMXRos staining

Mitochondria in hPSCs cultured on Matrigel-coated glass coverslips were visualized using Mitotracker Red CMXRos (MTT; Life Technologies) according to the manufacturer’s instructions. A 1 mM stock solution was diluted to 250 µM in DMEM/F12 and added to the cell culture medium to achieve a final concentration of 25 nM. Cells were incubated with the dye for 20 min at 37 °C.

Following staining, cells were washed five times with pre-warmed DMEM/F12, then transferred to ice, washed three times with 1× PBS, and fixed with 4% paraformaldehyde for 30 min on ice in the dark. After fixation, coverslips were mounted for imaging.

Fluorescence images were captured using a Zeiss LSM700 confocal microscope equipped with a ×40 1.3 oil differential interference contrast (DIC) lens (Carl Zeiss, Oberkochen, Germany). Images were taken from approximately equal-sized cell clusters using consistent acquisition settings.

### 2.9 Electron microscopy

CCTL14 hPSCs were seeded on Matrigel-coated dishes and cultured for 24 h under the respective treatment conditions. Cells were then harvested using TrypLE, washed with 1× PBS, fixed, and processed for transmission electron microscopy (TEM) as previously described (Moráň et al., 2019).

Ultrathin sections were prepared using a Leica EM UC6 ultramicrotome, stained with uranyl acetate and Reynolds’ lead citrate, and examined using an FEI Morgagni 286(D) transmission electron microscope (TEM).

### 2.10 Oxygen consumption measurement

To measure oxygen consumption dynamics, hPSCs were cultured in a monolayer on Matrigel-coated dishes containing 2 mL of CM + or CM-. Oxygen levels were continuously monitored using miniaturized Clark-type sensors (BVT Technologies, Brno, Czech Republic) with a polypropylene membrane, mounted on exchangeable holders. The measuring part of each sensor was embedded into the Petri dish lid at a distance of approximately 2 mm above the cell layer.

Two independent sensors were used simultaneously: one for cells cultured in CM+ and one for cells in CM-. Both sensors were connected to a QuadStat EA164 electrochemical analyzer (eDAQ, Denistone East, Australia), with a working potential of −650 mV versus an Ag/AgCl reference electrode. Data were acquired at 1-second intervals using Chart software (version 5.5.16) from eDAQ.

Prior to measurements, sensors were calibrated using sodium sulphite solutions. After calibration, sensors were washed with distilled water and 70% ethanol, placed into fresh cultivation medium, and allowed to stabilize for 30 min before insertion into Petri dishes containing cells. All measurements were conducted at 37 °C and atmospheric pressure (101 kPa).

Oxygen concentration in the culture medium (Om, in µM) was calculated using the following equation: Om = (Cm/Cw) × Ow. Where: Cm = measured current in the sample (mA), Cw = current measured in distilled water at equilibrium (mA), Ow = concentration of oxygen in distilled water under water-saturated air at 101 kPa and 37 °C, equal to 212 µM (according to IUPAC tables).

Linear regression of Om values over time was performed, and the slope of the regression line was used to determine the oxygen consumption rate.

### 2.11 Liquid chromatography coupled with mass spectrometry (LC-MS)

hPSCs cultured under 21% or 5% O_2_ were first deprived of FGF2 for 24 h, then treated with FGF2 (10 ng/mL) for 24 and an additional 2 h. After treatment, cells were washed with ice-cold PBS on ice (4 °C), collected in LC-MS buffer (60% acetonitrile, 30% methanol, 10% H_2_O), freeze-lysed at −80 °C, and analyzed within 2 h.

All reagents were of analytical grade, except for methanol and acetonitrile, which were LC-MS grade. Water was ultrapure, supplied by an in-house Milli-Q system (Millipore, MA, USA). Metabolite standards, acetonitrile (ACN), methanol (MeOH), acetic acid, formic acid, and ammonium hydroxide were purchased from Sigma-Aldrich (Prague, Czech Republic).

Metabolite standards were dissolved in water to prepare stock solutions (0.1 mg/mL), which were stored at −80 °C. Working standard solutions were prepared by diluting the stock solutions in ACN to a final metabolite concentration of 10 μg/mL. These standards were used to determine LC-MS parameters (retention time, peak shape, MS/MS fragmentation, and sensitivity), and were compared to the chromatographic profiles of cell lysates.

Biological replicates (N = 3) were processed by mixing cell pellets with 1 mL of 90% ACN, followed by a short centrifugation step. Twenty microliters of the supernatant were injected onto the analytical column.

The LC-MS system consisted of a Dionex Ultimate 3000RS (Thermo Scientific, CA, USA) equipped with a binary high-pressure gradient pump, autosampler, and column oven. Metabolites were separated using a SeQuant ZIC-cHILIC analytical column (100 × 2.1 mm, 3 μm), with a matching guard column (2 × 2.1 mm, 3 μm). The mobile phase consisted of 90% ACN (solvent A) and 100 mM ammonium formate in water (solvent B). A linear gradient from 90% A to 50% A over 15 min was followed by a 1-min hold, then a 1-min re-equilibration at initial conditions. The flow rate was 0.3 mL/min, and the column temperature was maintained at 23 °C ± 0.1 °C.

The LC system was coupled to a EVOQ Qube triple quadrupole mass spectrometer (Bruker, Germany), operated in positive or negative heated electrospray ionization (HESI) mode. The connection was made via a divert valve and PEEK capillary. Instrument parameters were as follows: Spray voltage: +4000 V/−3500 V, Cone temperature: 350 °C, Cone gas: 20 psi, Heated probe temperature: 300 °C, Probe gas flow: 40 psi (nitrogen), Nebulizer gas: 45 psi (nitrogen), Collision gas: Argon.

Flow was diverted to waste during 0–1 min and 16.5–25 min of the run. Metabolites were detected in selected reaction monitoring (SRM) mode, targeting fragments of [M + H]^+^ or [M–H]^-^ ions (details in Supplementary Figure S7).

Metabolite concentrations were normalized to the total metabolite content in each sample and relativized to FGF2-treated cells under 21% O_2_ conditions.

### 2.12 Statistical analysis

The number of independent biological replicates (N) is indicated in the respective figure legends. Arithmetic means, standard error of the mean (SEM), and statistical analyses were calculated using GraphPad Prism 8 (GraphPad Software, La Jolla, CA, USA).

In the figure legends, N = number of independent replicates. Statistical significance was assessed using one-sample t-test (theoretical mean = 1) for comparisons against control samples used for normalization and paired two-tailed t-test for direct comparisons between experimental conditions. Exceptions to these tests or variations in how data are presented are specified in the corresponding figure legends. Raw values used to generate all graphs and associated statistics are available in the shared dataset (https://doi.org/10.6084/m9.figshare.29651828).

Statistical significance is indicated as follows: *p* < 0.05 (*), p < 0.01 (**), p < 0.001 (***).

## 3 Results

### 3.1 FGF2 decreases PDH phosphorylation and increases histone acetylation and nanog levels in 5% O_2_

**FIGURE 1:**
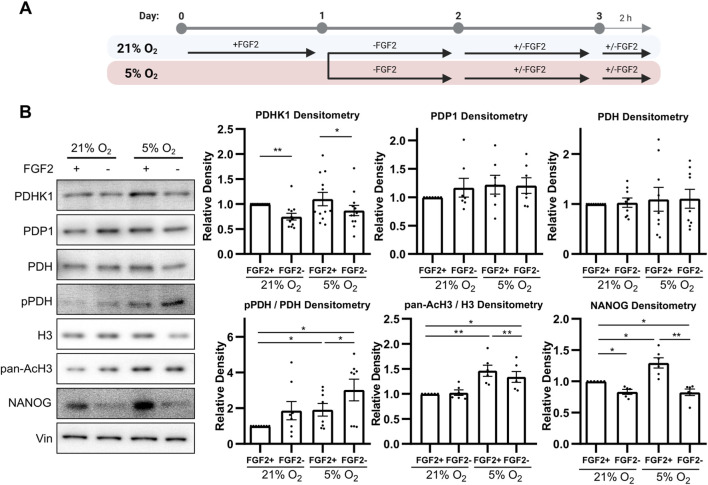
FGF2 activates pyruvate dehydrogenase and increases histone acetylation and NANOG levels in 5% O_2_ **(A)** Diagram of the experimental workflow. **(B)** Representative Western blots and corresponding densitometric analyses of PDHK1 (N = 12), PDP1 (N = 7), total PDH (N = 9), the ratio of phosphorylated to total PDH (N = 8), the ratio of pan-acetylated histone H3 to total H3 (AcH3; N = 6), and NANOG (N = 6). FGF2 treatment under 5% O_2_ resulted in a significant decrease in PDH phosphorylation and a marked increase in PDHK1, AcH3, and NANOG levels. Vinculin (Vin) was used as a loading control. N = number of independent replicates.

To investigate how PDH activity is regulated, we analyzed the protein levels of pyruvate dehydrogenase kinase 1 (PDHK1), pyruvate dehydrogenase phosphatase 1 (PDP1), total PDH, and PDH phosphorylation at serine 293 (pPDH)—its most rapidly and abundantly phosphorylated site targeted by all isoforms of PDHKs (Rardin et al., 2009; Yeaman et al., 1978) using Western blot (WB). To study the effect of FGF2 under different oxygen conditions, hPSCs were cultured in either 21% or 5% O_2_ and treated with FGF2 (10 ng/mL). Cells were starved of FGF2 for 24 h, then treated with FGF2 for 24 h, followed by a media change and a second 2-h FGF2 treatment (Figure 1A). This protocol was used to capture both immediate and delayed effects of FGF2 signaling, as FGF2 is thermolabile and rapidly loses activity at 37 °C (Chen et al., 2012). Cells not treated with FGF2 served as negative controls.

Under 5% O_2_, FGF2 treatment significantly decreased PDH phosphorylation and simultaneously increased PDHK1 protein levels compared to untreated cells, while PDP1 levels remained unchanged (Figure 1B). A similar trend in PDHK1 and PDP1 expression was observed under 21% O_2_; however, the decrease in PDH phosphorylation did not reach statistical significance. Overall, PDH phosphorylation levels were significantly higher under 5% O_2_ than in the corresponding conditions at 21% O_2_, in line with metabolic adaptation to low oxygen levels.

To assess changes in histone acetylation, we performed WB for pan-acetylated histone H3 (AcH3) using an antibody recognizing five acetylation sites (K9, K14, K18, K23, and K27). In 5% O_2_, AcH3 levels were significantly higher compared to 21% O_2_ and FGF2 treatment significantly increased AcH3 levels (Figure 1B). In contrast, no increase in AcH3 was observed in FGF2-treated cells under 21% O_2_.

Next, we evaluated whether this increase in histone acetylation correlates with levels of NANOG, because NANOG is the most variable pluripotency marker (Hayashi et al., 2019; Kalmar et al., 2009; M. Li and Izpisua Belmonte, 2018) which was shown to specifically depend on histone acetylation (Hattori et al., 2007; Horne et al., 2014; M. S. Kim et al., 2015). FGF2 treatment led to a significant increase in NANOG levels under both oxygen conditions (Figure 1B). However, NANOG expression was significantly higher in cells treated with FGF2 under 5% O_2_ compared to 21% O_2_.

Since PDH and its regulatory enzymes reside in mitochondria—and mitochondria in hPSCs are typically small, perinuclearly localized, and have underdeveloped cristae (Choi et al., 2015)—we assessed the effect of FGF2 on mitochondrial morphology. Cells cultured under 21% O_2_ were stained with MitoTracker Red CMXRos or analyzed by transmission electron microscopy following 24-h FGF2 treatment or starvation. No morphological changes in mitochondrial size or cristae development were observed (Supplementary Figure S1). However, treatment with high dose of dichloroacetate (DCA, 20 mM for 24 h), an inhibitor of PDHK1, led to mitochondrial elongation and cristae development (Supplementary Figure S1). FGF2 had no apparent effect on this process.

### 3.2 PDH-mediated Acetyl-CoA production is essential for histone acetylation and NANOG expression

**FIGURE 2:**
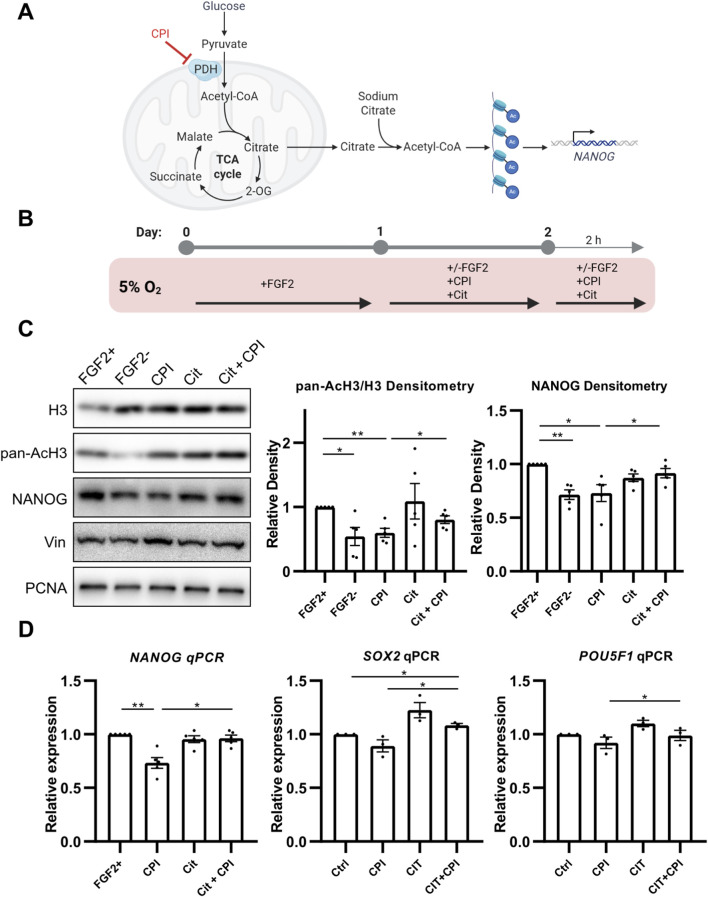
PDH activity promotes histone H3 acetylation via Ac-CoA production, enhancing NANOG expression. **(A)** Schematic representation of the treatment strategy. The small-molecule inhibitor CPI suppresses PDH activity, resulting in reduced Ac-CoA production, decreased histone acetylation, and lower NANOG expression. These effects can be rescued by the addition of sodium citrate, an Ac-CoA precursor. **(B)** Diagram of the experimental workflow. **(C)** Representative Western blots and densitometric analyses of the ratio of pan-acetylated histone H3 to H3 (AcH3; N = 5) and NANOG (N = 5) following PDH inhibition by CPI-613 (CPI) and rescue by sodium citrate (Cit). CPI treatment significantly reduced AcH3 and NANOG levels to those of the cells without FGF2, while sodium citrate co-treatment restored their expression. Vinculin (Vin) and PCNA were used as loading controls. N = number of independent replicates. **(D)** Quantitative PCR analysis of the pluripotency markers *NANOG* (N = 5), *SOX2* (N = 3), and *POU5F1* (N = 3). CPI treatment significantly downregulated *NANOG* and showed a non-significant trend toward reduced *SOX2* and *POU5F1* expression. Co-treatment with sodium citrate significantly increased expression levels of all three pluripotency markers compared to CPI treatment. Gene expression was normalized to GAPDH. N = number of independent replicates.

As PDH produces the glycolysis-derived Ac-CoA used for histone acetylation (Figure 2A), we hypothesized that FGF2 might increase PDH activity in 5% O_2_ to support this process and consequently upregulate NANOG levels. To test this, we inhibited PDH using the small molecule inhibitor CPI-613 (CPI; 10 μM) (Smith and Hewitson, 2020) for 24 h, followed by an additional 2-h treatment after media change (Figure 2B). PDH inhibition by CPI led to a significant reduction in pan-acetylation of histone H3, and NANOG protein level (Figure 2C). We also specifically assayed the acetylation of H3K9 and H3K27 which are strongly associated with transcriptional activation and dynamic changes during metabolic reprogramming (A. M. Li et al., 2023; Y. Li et al., 2020). CPI decreased acetylation of both (Supplementary Figure S2). PDH inhibition also led to significant downregulation of *NANOG* expression and similar, statistically insignificant trend in *SOX2* and *POU5F1* expression (Figure 2D).

These results suggest that PDH activation by FGF2 is required to maintain histone acetylation and NANOG levels in hPSCs under 5% O_2_. To test whether PDH-derived Ac-CoA is indeed essential for these effects, we attempted to rescue the impact of PDH inhibition by supplementing cells with 5 mM sodium citrate, a metabolite that bypasses PDH-mediated Ac-CoA production, replenishing it in cytosol (Petillo et al., 2020) (Figures 2A,B). Indeed, sodium citrate treatment significantly restored pan-acetylation of H3, acetylation of H3K9 (Supplementary Figure S2), and NANOG levels and *NANOG*, *SOX2*, and *POU5F1* expression in CPI-treated cells (Figures 2C,D). The rescue of H3K27 acetylation was only minute and not statistically significant (Supplementary Figure S2). We validated this experiment using sodium acetate instead of sodium citrate and observed similar trends (Supplementary Figure S2). Interestingly, sodium citrate treatment alone increased the expression of both *SOX2* and *POU5F1*, albeit without statistical significance.

To determine whether PDH-mediated Ac-CoA production and the resulting changes in pluripotency markers expression are key drivers of FGF2-induced pluripotency, we activated PDH in FGF2-deprived cells using the PDHK inhibitor DCA. Treatment with a range of DCA concentrations (0.5–5 mM) effectively reduced PDH phosphorylation, indicating increased enzymatic activity (Supplementary Figure S3). However, this activation did not lead to significant changes in global H3 acetylation or NANOG levels.

Taken together, these findings confirm that, in addition to previously described effects of FGF2 on pluripotency markers expression, FGF2-mediated activation of PDH in 5% O_2_ constitutes a key mechanism for generating the Ac-CoA pool required for histone acetylation. Moreover, they demonstrate that NANOG expression in hPSCs cultured under 5% O_2_ is dependent on this metabolic pathway. A schematic representation of this regulation is depicted in Figure 2A.

### 3.3 FGF2 downregulates reactive oxygen species in 5% O_2,_ increasing redox-sensitive PDH activity

**FIGURE 3:**
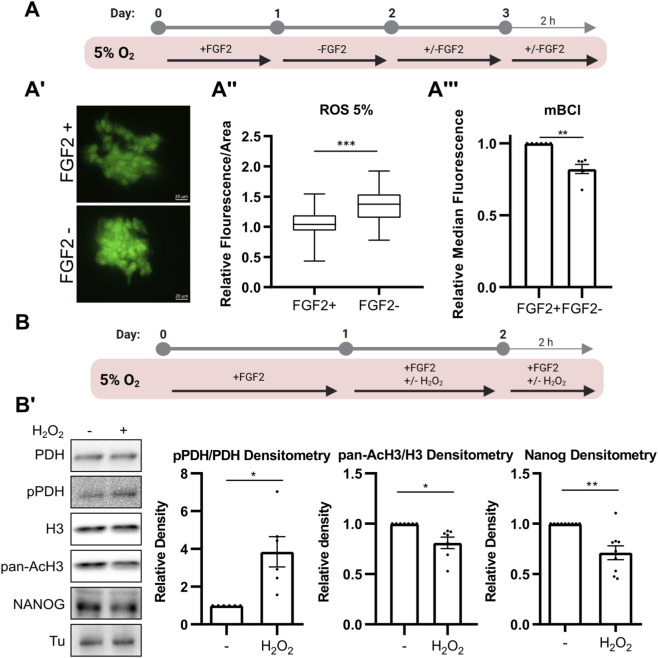
FGF2 reduces ROS levels via MEK1/2-ERK1/2 pathway, thereby limiting PDH phosphorylation and maintaining AcH3 and NANOG expression. **(A)** Schematic of the experimental workflow for ROS and GSH quantification. **(A′)** Representative images of CellROX Green fluorescence in hPSCs cultured with or without FGF2. Scale bars = 20 µm. **(A″)** Quantification of ROS levels in hPSCs cultured at 5% O_2_ using CellROX Green. Signal intensity per area was calculated, and values were relativized to the average of FGF2-treated (FGF2+) cells within each biological replicate. Data are compiled from five independent experiments (≥10 measurements per condition; FGF2+ N = 70; FGF2- N = 67). N refers to the total number of analyzed images. FGF2 significantly reduced ROS levels. Statistical significance was determined using an unpaired two-tailed t-test. **(A‴)** Measurement of intracellular reduced glutathione (GSH) levels using monochlorobimane (mBCl) and flow cytometry. FGF2 increases reduced GSH levels. Median fluorescence values were relativized to FGF2+ cells (N = 6). N = number of independent replicates. **(B)** Schematic of the experimental workflow for analyzing the effect of ROS on PDH phosphorylation, AcH3, and NANOG levels. **(B′)** Representative Western blot and densitometric quantification of PDH phosphorylation (pPDH/PDH; N = 6), pan-acetylated H3 (AcH3; N = 7), and NANOG (N = 9) following H_2_O_2_ treatment (5 μM, 24 + 2 h). ROS elevation led to increased PDH phosphorylation and decreased AcH3 and NANOG levels. Tubulin (Tu) was used as a loading control. N = number of independent replicates.

We previously reported that culturing hPSCs at 5% O_2_ reduces levels of ROS—key second messengers known to modulate FGF2 signaling—and that FGF2 signaling likely contributes to ROS suppression in 5% O_2_, as MEK1/2 inhibition led to increased ROS levels under 5% but not 21% O_2_ (Fojtík et al., 2021). To confirm this, hPSCs were either deprived of FGF2 or treated with FGF2 (10 ng/mL) for 24 h, followed by an additional 2-h treatment after media change in 5% O_2_ (Figure 3A). ROS levels were assayed using CellROX Green fluorescent probe (5 µM for 50 min). Signal intensity was measured from snapshots taken by a fluorescent microscope (Figure 3A’). Consistent with our previous findings on MEK1/2, we show that FGF2 significantly lowers ROS levels in hPSCs cultured at 5% O_2_ (Figure 3A’’). To complement ROS quantification, we measured intracellular levels of reduced GSH—the principal cellular antioxidant—using the mBCl fluorescent probe and flow cytometry using the same treatment plan (Figure 3A). In line with elevated ROS in FGF2-starved cells, we observed a statistically significant decrease in reduced GSH levels (Figure 3A).

The FGF2-induced increase in histone H3 acetylation observed exclusively under 5% O_2_ conditions (Figure 1B) suggests, that this mechanism is oxygen-sensitive. Since FGF2 downregulates ROS in 5% O_2_, we have previously shown lower ROS levels in 5% compared to 21% O_2_ (Fojtík et al., 2021), and PDH can be regulated by ROS (Cesi et al., 2017), we wanted to investigate whether it could be also ROS-sensitive. To do so, we elevated ROS levels in hPSCs cultured at 5% O_2_ by treating them with 5 μM hydrogen peroxide (H_2_O_2_) for 24 h, followed by an additional 2 h post-media change (Figure 3B). H_2_O_2_-treated cells showed a significant increase in PDH phosphorylation, along with a marked decrease in H3 pan-acetylation and NANOG levels (Figure 3B’). We also observed a significant decrease in H3K9 acetylation and a minor, statistically insignificant decrease in H3K27 acetylation (Supplementary Figure S4).

Next, we investigated whether PDP1 is regulated by ROS, given that both protein phosphatases (Sommer et al., 2002; Wright et al., 2009) and mitochondrial enzymes (Napolitano et al., 2021) are known to be sensitive to ROS-mediated modulation. Moreover, PDP1 has recently been implicated in the regulation of histone acetylation (Karagiota et al., 2022). Since we observed a more pronounced effect of FGF2 on PDH phosphorylation under low-ROS conditions (5% O_2_) compared to 21% O_2_ (Figure 1B), we first examined whether PDP1 activity is differentially regulated across these oxygen conditions.

To assess PDP1 activity, we used esiRNA to silence *PDP1* expression and measured the resulting changes in PDH phosphorylation compared to control cells. Knockdown of PDP1 significantly increased PDH phosphorylation at 5% O_2_, whereas only a partial effect was observed in cells cultured at 21% O_2_ (Supplementary Figure S5). To determine whether this difference could be attributed to ROS, we treated PDP1-silenced cells at 21% O_2_ with GSH (5 mM, 1 h), a well-established antioxidant. GSH treatment led to a significant increase in PDH phosphorylation in PDP1-silenced cells compared to the corresponding untreated control (Supplementary Figure S5), suggesting that endogenous ROS levels at 21% O_2_ are sufficient to reversibly inhibit PDP1. Additionally, GSH treatment of control (non-silenced) cells resulted in reduced PDH phosphorylation relative to untreated controls, further supporting the role of ROS in modulating PDH activity. Finally, Western blot analysis of PDP1 under non-reducing conditions revealed a mobility shift in H_2_O_2_-treated cells, suggesting PDP1 oxidation (Supplementary Figure S5). However, this mobility shift could be also caused by other post-translational modifications.

### 3.4 FGF2 regulates PDH activity in 5% O_2_ by MEK1/2-ERK1/2 mediated downregulation of ROS

**FIGURE 4:**
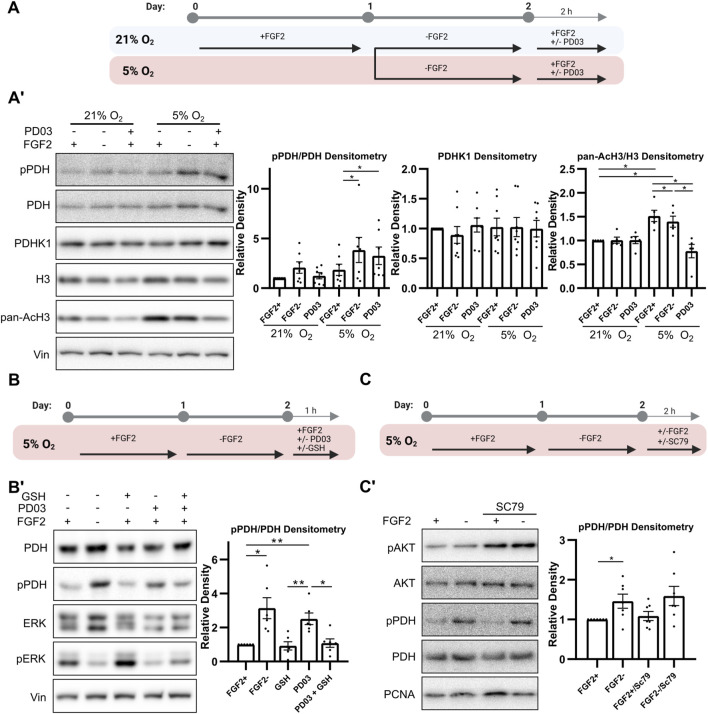
MEK1/2–ERK1/2 signaling reduces PDH phosphorylation via ROS downregulation. **(A)** Schematic of the experimental workflow for MEK1/2 inhibition by PD0325901 (PD03) in hPSCs cultured under 21% and 5% O_2_ conditions. **(A′)** Western blot analysis and densitometric quantification of phosphorylated PDH (pPDH; N = 7), pan-acetylated H3 (AcH3; N = 5), and PDHK1 (N = 8) levels following PD03 treatment. MEK1/2 inhibition significantly reduced AcH3 levels in both oxygen conditions, while it increased pPDH levels only under 5% O_2_. PDHK1 expression remained unaffected. Vinculin (Vin) served as a loading control. N = number of independent replicates. **(B)** Schematic of the experimental workflow for testing whether antioxidant treatment can rescue the effects of MEK1/2 inhibition. **(B′)** Western blot analysis of PDH phosphorylation following treatment with reduced glutathione (GSH; 5 mM, 1 h) in PD03-treated hPSCs under 5% O_2_ (N = 6). GSH attenuated the PDH hyperphosphorylation induced by MEK1/2 inhibition. PCNA was used as a loading control. N = number of independent replicates. **(C)** Schematic of the experimental workflow for AKT activation by SC79. **(C′)** Western blot analysis showing that SC79 treatment successfully increased AKT phosphorylation but had no effect on pPDH levels in 5% O_2_ (N = 7). PCNA was used as a loading control. N = number of independent replicates.

We have previously shown that FGF2-activated MEK1/2–ERK1/2 signaling reduces ROS levels in hPSCs in 5% O_2_ (Fojtík et al., 2021). Here, we tested whether the inhibition of this pathway, leading to increased ROS levels, affects PDH phosphorylation as we show ROS-mediated regulation of PDH (Figure 3B’) and it was also reported in melanoma cells (Cesi et al., 2017). Furthermore, we analyzed whether it subsequently influences histone acetylation. To this end, we treated hPSCs with the MEK1/2 inhibitor PD0325901 (PD03; 0.2 μM for 2 h) under 21% and 5% O_2_ (Figure 4A) and observed oxygen-dependent effects. MEK1/2 inhibition in 21% O_2_ did not significantly affect PDH phosphorylation (Figure 4A’). In contrast, in 5% O_2_ it led to a significant increase in PDH phosphorylation, reaching levels similar to those observed in FGF2-starved cells. The increase in PDH phosphorylation under 5% O_2_ was accompanied by a significant reduction in mitochondrial membrane potential (Supplementary Figure S6). This suggests decreased activity of TCA cycle which is in line with PDH inactivation, however, mitochondrial membrane potential can be maintained by alternative mechanisms as well. Interestingly, MEK1/2 inhibition led to a decrease in pan-acetylation of H3 in 5% O_2_. Notably, PDHK1 levels remained unchanged regardless of experimental conditions.

To determine whether ROS upregulation underlies the increased PDH phosphorylation following MEK1/2 inhibition, we co-treated cells with PD03 (0.2 μM for 1 h) and the antioxidant GSH (5 mM for 1 h) in 5% O_2_ (Figure 4B). GSH treatment suppressed the PD03-induced increase in PDH phosphorylation (Figure 4B’), suggesting that MEK1/2–ERK1/2 signaling maintains low PDH phosphorylation in part by reducing ROS.

To distinguish the role of ROS in regulating PDH phosphorylation from the concurrent ROS-mediated AKT activation (Fojtík et al., 2021), we treated FGF2-starved cells (24 h) cultured at 5% O_2_ with the AKT activator SC79 (10 μM for 2 h), alongside FGF2 (10 ng/mL for 2 h) (Figure 4C). Although SC79 treatment increased AKT phosphorylation, it had no effect on PDH phosphorylation (Figure 4C’), supporting the conclusion that ROS—but not AKT—are involved in regulating PDH phosphorylation in this context. Phospho-AKT densitometry is shown in Supplementary Figure S6.

### 3.5 Metabolic readouts confirm functional activation of PDH by FGF2 at 5% O_2_

**FIGURE 5:**
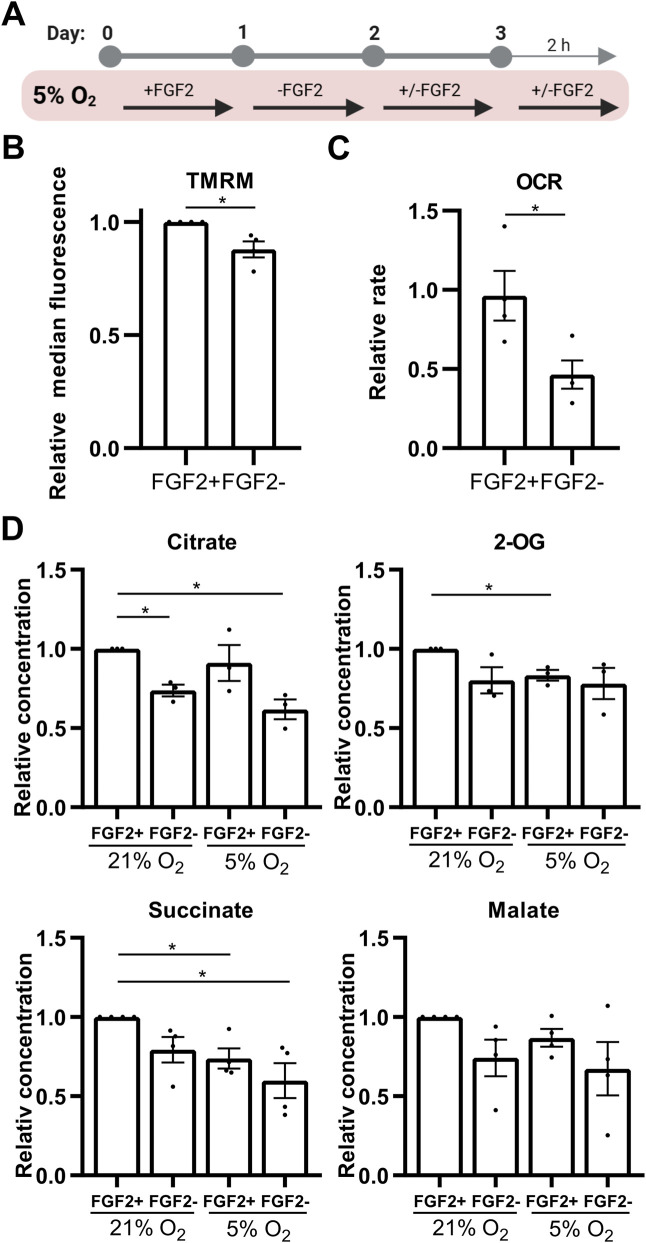
FGF2 enhances oxidative phosphorylation and diverts citrate from the TCA cycle in hPSCs cultured under 5% O_2_. **(A)** Schematic overview of the experimental workflows used to assess mitochondrial function, oxygen consumption, and metabolite changes. **(B)** Quantification of mitochondrial membrane potential in 5% O_2_ using TMRM staining and flow cytometry. FGF2 treatment significantly increased mitochondrial membrane potential. Data are shown as median fluorescence intensity relativized to control (±SEM, N = 4). N = number of independent replicates. **(C)** Oxygen consumption rate (OCR) measurements in 5% O_2_. FGF2-treated cells exhibited a significant increase in OCR. Data are presented as relativized values (nM O_2_/million cells/min) ± SEM (N = 4). Statistical significance was assessed using a two-tailed unpaired t-test with unequal variances. N = number of independent replicates. **(D)** Quantification of selected TCA cycle metabolites in hPSCs cultured under 21% and 5% O_2_. FGF2 treatment led to an increase in citrate levels (N = 3) under both oxygen conditions, whereas levels of two-oxoglutarate (2-OG; N = 3) and succinate (N = 4) decreased in 5% O_2_. A similar decreasing trend was observed for malate (N = 4). N = number of independent replicates.

To further confirm that unphosphorylated PDH is functionally active in FGF2-treated hPSCs at 5% O_2_, we assessed mitochondrial function using multiple parameters: mitochondrial membrane potential (as a readout of electron transport chain activity), oxygen consumption rate, and TCA cycle metabolite concentrations. Cells were treated with FGF2 (10 ng/mL) for 24 h and again for 2 h following a media change and compared to FGF2-starved controls (Figure 5A). FGF2 treatment led to a modest but statistically significant increase in mitochondrial membrane potential (Figure 5B), which correlated with significantly elevated oxygen consumption as measured using a Clark-type electrode (Figure 5C). These findings are consistent with increased PDH activity. Furthermore, FGF2 differentially affected TCA cycle metabolite levels depending on oxygen availability. In 5% O_2_, citrate levels in FGF2-treated cells were comparable to those observed in FGF2-treated cells at 21% O_2_, but were elevated relative to cells cultured without FGF2, consistent with reduced PDH activity (Figure 5D). Interestingly, downstream TCA intermediates—2-oxoglutarate (2-OG) and succinate—were significantly lower in FGF2-treated cells at 5% O_2_ compared to the same treatment at 21% O_2_, suggesting that citrate does not fully propagate through the TCA cycle in these conditions. However, citrate can be also produced by reductive carboxylation of 2-OG. Together, these data suggest that PDH activity is higher in FGF2-treated hPSCs at 5% O_2_, in line with our observations of reduced PDH phosphorylation (Figure 1B).

## 4 Discussion

### 4.1 FGF2 promotes the use of the TCA cycle under low oxygen in hPSCs to supply Ac-CoA for histone acetylation and upregulate NANOG

Cells generate most of their ATP from glucose, which is first metabolized via glycolysis and subsequently through the TCA cycle and OxPhos in mitochondria. Under low oxygen conditions, however, cells primarily rely on glycolysis, which does not require oxygen for ATP production. A key metabolic switch between glycolysis alone and glycolysis coupled to OxPhos is PDH, which converts pyruvate—the end product of glycolysis—into Ac-CoA, the entry metabolite of the TCA cycle.

Consistent with this model, we observed an increase in phosphorylated (inactive) PDH in hPSCs cultured at 5% O_2_ compared to 21% O_2_, indicating reduced TCA cycle activity and mitochondrial respiration under low oxygen conditions. This aligns with canonical adaptations to hypoxia (Formenti and Constantin-Teodosiu, 2010; Goda and Kanai, 2012). Interestingly, treatment with FGF2 led to increased levels of unphosphorylated (active) PDH in 5% O_2_, accompanied by elevated mitochondrial membrane potential, oxygen consumption, and citrate levels. These results suggest that FGF2 stimulates partial engagement of the TCA cycle and OxPhos even under hypoxic conditions.

Our observation that citrate levels did not fully propagate through the TCA cycle in FGF2-treated cells cultured in 5% O_2_ suggested that a portion of citrate may be diverted away from energy metabolism. Since citrate can exit mitochondria via the citrate shuttle and be cleaved to regenerate cytosolic Ac-CoA, we hypothesized that it may be redirected toward other processes such as histone acetylation (Jo et al., 2020; Moussaieff et al., 2015; Wellen et al., 2009).

Ac-CoA is an indispensable substrate for histone acetylation (Jo et al., 2020), an epigenetic modification recognized as a hallmark of pluripotency (Huang et al., 2016; Mu et al., 2015; Trisciuoglio et al., 2018). Furthermore, in hPSCs, glucose-derived Ac-CoA has been identified as the primary source of substrate for this process (Moussaieff et al., 2015). Therefore, we explored whether FGF2-driven activation of PDH and the resulting increase in TCA activity under 5% O_2_ promotes histone acetylation. Consistent with this hypothesis, we observed a marked decrease in histone acetylation upon PDH inhibition and rescue of this effect after treatment with sodium citrate and sodium acetate to bypass the PDH-mediated Ac-CoA production and the citrate shuttle. Aside from pan-acetylation of H3, we analyzed acetylation of H3K9 and H3K27, which are strongly associated with transcriptional activation and undergo dynamic changes during metabolic reprogramming (A. M. Li et al., 2023; Y. Li et al., 2020). Interestingly, we observed strong effect of FGF2 treatment and PDH activity under 5% O_2_ on acetylation of H3K9 and only minor effect on H3K27. Based on that we speculate that only H3K9-specific acetyltransferases, like Gcn5 (Jin et al., 2011), are influenced by this FGF2-induced metabolic regulation in hPSCs.

To further explore whether the PDH-mediated Ac-CoA production reinforces pluripotency, we analyzed the expression of three core pluripotency transcription factors: *NANOG*, *SOX2*, and *POU5F1* (encoding OCT-4). Inhibition of PDH led to a decrease in the expression of all three genes (albeit insignificantly for *SOX2* and *POU5F1*), while supplementation with an Ac-CoA precursor sodium citrate rescued their expression with statistical significance. Notably, *NANOG* expression was most strongly affected, which we confirmed at the protein level. This is particularly relevant, as NANOG protein levels are known to oscillate the most in hPSCs (Hayashi et al., 2019; Kalmar et al., 2009; M. Li and Izpisua Belmonte, 2018).

The relatively modest changes observed in *SOX2* and *POU5F1* expression following PDH inhibition may reflect their different sensitivity to histone acetylation, as suggested by their differential responses to citrate treatment. This is supported by the fact that expression of *NANOG*, but not *SOX2* and *POU5F1*, seems to be particularly dependent on histone acetylation (Hattori et al., 2007; Horne et al., 2014; M. S. Kim et al., 2015). Notably, NANOG, SOX2, and OCT-4 are known to participate in a positive regulatory feedback loop (Pan and Thomson, 2007), implying that the downregulation of one may secondarily influence the expression of the others. Therefore, we cannot distinguish whether the limited impact of PDH inhibition on *SOX2* and *POU5F1* expression is a direct consequence of reduced histone acetylation, or an indirect effect mediated by decreased NANOG levels.

Taken together, our data demonstrate that PDH activity is essential for histone acetylation and *NANOG* expression in hPSCs under low oxygen conditions, consistent with findings in other cell types (Sutendra et al., 2014). We therefore propose that the observed increases in oxygen consumption and mitochondrial membrane potential which were induced by FGF2 are byproducts of enhanced PDH-mediated Ac-CoA production, which supports histone acetylation (Moussaieff et al., 2015) and, consequently, expression of *NANOG* (Hattori et al., 2007; Horne et al., 2014; M. S. Kim et al., 2015).

While the essential role of FGF2 in maintaining hPSC pluripotency is well established (Haghighi et al., 2018; Kjartansdóttir et al., 2012), our findings indicate that the FGF2-mediated increase in global histone H3 pan-acetylation is not the primary driver of this effect. Instead, histone acetylation appears to serve as a supportive mechanism within the broader network of FGF2-mediated pluripotency maintenance, likely enhancing but not solely dictating the expression of key pluripotency markers.

Interestingly, we did not observe significant effects of FGF2 on PDH activity or histone acetylation under 21% O_2_, suggesting that these mechanisms are specific to the low oxygen environment. This is in agreement with previous studies showing that 5% O_2_ enhances glycolysis which in turn promotes chromatin-opening epigenetic modifications (Karagiota et al., 2022; J. Kim et al., 2006; Lees et al., 2019; Moussaieff et al., 2015; Okazaki and Maltepe, 2006). One possible explanation for this oxygen-dependent effect is the involvement of HIFs, which promote glycolysis (Papandreou et al., 2006; Semenza, 2011) and have been implicated in regulating H3 acetylation in hPSCs (Cui et al., 2020). Alternatively, 5% O_2_ and FGF2 signaling might stimulate nuclear translocation of PDH implicated in regulation of histone acetylation (Nagaraj et al., 2017; Sutendra et al., 2014).

### 4.2 PDH phosphorylation is downregulated via FGF2-MEK1/2-ERK1/2-ROS axis

Interestingly, we observed decreased PDH phosphorylation following FGF2 treatment in 5% O_2_, despite detecting increased levels of its kinase PDHK1 and unchanged levels of its phosphatase PDP1. These findings suggest that the regulation of PDH phosphorylation does not occur at the transcriptional level, as it does, for example, with TGF-β1 in fibroblasts (Smith and Hewitson, 2020). Instead, the activity of these enzymes appear to be modulated post-translationally, and this regulation is oxygen-dependent according to our results.

Combining our findings from this study with our previous work (Fojtík et al., 2021), we demonstrate that FGF2 reduces intracellular ROS levels in 5% O_2_ via MEK1/2-ERK1/2 signaling. While traditionally viewed as damaging byproducts of metabolism, ROS also function as important signaling molecules that influence various cellular pathways (Sies and Jones, 2020; Sinenko et al., 2021). Importantly, ROS have been shown to regulate key enzymes in glycolysis and oxidative metabolism (Liemburg-Apers et al., 2015; Molavian et al., 2016), including promoting PDH phosphorylation (Cesi et al., 2017). Based on these insights, we hypothesized that ROS mediate the effect of FGF2 on PDH activity in 5% O_2_. Supporting this, we found that elevating ROS levels within a physiological range increases PDH phosphorylation and concurrently reduces histone acetylation and NANOG protein levels—mimicking the phenotype observed in FGF2-deprived cells. Furthermore, we show that FGF2 suppresses PDH phosphorylation through MEK1/2-ERK1/2-dependent ROS downregulation in 5% but not in 21% O_2_, which is in concert with our previous data showing that MEK1/2 inhibition increases ROS levels only in 5% O_2_ (Fojtík et al., 2021). These data identify ROS as a key mediator linking FGF2 signaling to PDH activity and downstream epigenetic and transcriptional effects.

Dissecting the precise mechanism of MAPK-mediated ROS attenuation is beyond the scope of this study. However, one possibility is that ERK1/2 phosphorylates HIFs, enhancing their transcriptional activity (Mylonis et al., 2008; Richard et al., 1999), leading to upregulation of glycolysis, which in turn promotes the production of glutathione (GSH) and NADPH—key components of the cellular antioxidant defense system (Samanta and Semenza, 2017). This is consistent with our previous observation that MEK1/2-ERK1/2-dependent ROS suppression is specific to hPSCs cultured in 5% O_2_ (Fojtík et al., 2021). Furthermore, the increased levels of reduced GSH detected in FGF2-treated cells support this hypothesis.

Although our data demonstrate that the MEK1/2–ERK1/2 pathway regulates PDH phosphorylation via ROS, it appears to influence histone acetylation through additional, ROS-independent mechanisms. This is supported by our observation that MEK1/2 inhibition reduces AcH3 levels more strongly than FGF2 deprivation alone. Mechanistic links between MEK1/2–ERK1/2 signaling and histone acetylation have been extensively studied, particularly in the context of memory formation (Levenson et al., 2004). One proposed mechanism involves ERK1/2-mediated phosphorylation of histones, which facilitates the recruitment of the Gcn5 histone acetyltransferase (Cheung et al., 2000). This would be in line with our observation that the acetylation of H3K9 strongly correlates with FGF2 treatment and activity of PDH, as Gcn5 is considered the primary histone acetyltransferase for H3K9 (Jin et al., 2011).

We have also previously shown that elevated ROS can activate the PI3K/AKT pathway in hPSCs (Fojtík et al., 2021), and others have reported that AKT activation may contribute to PDH phosphorylation (Cerniglia et al., 2015), consistent with its role in promoting glycolytic adaptation to hypoxia (Xie et al., 2019). However, in our experiments, activation of AKT by a pharmacological agonist did not increase PDH phosphorylation in hPSCs cultured at 5% O_2_. This indicates that FGF2-mediated regulation of PDH phosphorylation operates exclusively through the MAPK pathway in this context.

### 4.3 Susceptibility of PDP1 to ROS

While ROS-induced phosphorylation of PDH has been previously observed (Cesi et al., 2017), the molecular mechanism underlying this effect remains unclear. In our study, we noted that PDH phosphorylation remains low in FGF2-treated cells cultured at 5% O_2_, despite elevated levels of its kinase, PDHK1. This led us to hypothesize that the phosphatase PDP1 might play a role in this regulation. This would be in line with a recent study reporting that PDP1-mediated activation of PDH is required for histone acetylation under hypoxic conditions in cancer cell lines (Karagiota et al., 2022). Although we did not observe changes in PDP1 protein levels, it is known that many mitochondrial enzymes are sensitive to ROS (Napolitano et al., 2021), and protein phosphatases are particularly prone to reversible oxidation (Chiarugi and Cirri, 2003; Meng et al., 2002; Östman et al., 2011; Sommer et al., 2002).

PDP1 belongs to the protein phosphatase 2C family of Ser/Thr phosphatases, which are generally described as having low or negligible sensitivity to oxidation (Sommer et al., 2002). However, oxidation of these enzymes has been observed under certain conditions (Pieri et al., 2003), and it has been proposed that prior failures to detect this were due to the use of ambient oxygen conditions (21%) in experimental settings (Wright et al., 2009), which can suppress Ser/Thr phosphatase activity (Nyunoya et al., 2005).

Our data support a model in which PDP1 is sensitive to oxygen and ROS levels. Notably, we observed an oxygen- and ROS-dependent effect on PDP1 activity and a mobility shift of PDP1 in native gel electrophoresis following H_2_O_2_ treatment, suggesting its oxidation. Nevertheless, we cannot exclude the possibility that the observed shift in PDP1 mobility reflects other post-translational modifications, and thus our study does not provide direct evidence that PDP1 undergoes reversible oxidation.

### 4.4 Physiological relevance, experimental controls, and limitations

It is important to note that although histone acetylation is often associated with pluripotency (Huang et al., 2016; Mu et al., 2015; Trisciuoglio et al., 2018), it is not a definitive marker of either pluripotency or differentiation. Rather, histone acetylation facilitates transcriptional activation by promoting chromatin accessibility, making its functional outcome highly context-dependent. For instance, under severe hypoxic conditions (0.5%–1% O_2_), which resemble oxygen levels found in tumors, PDHK1 induction and PDH phosphorylation can reduce histone acetylation, thereby silencing neuronal differentiation markers in neuroblastoma cells (Y. Li et al., 2020).

Chemical induction of ROS carries the risk of causing unphysiological oxidative stress. To avoid this, we used a low concentration of H_2_O_2_ (5 μM), which corresponds to the upper limit of H_2_O_2_ levels reported in human plasma (Forman et al., 2016). Intracellular H_2_O_2_ concentrations are typically estimated to range between 1 and 10 nM (Lyublinskaya and Antunes, 2019; Sies, 2017). Due to the limited permeability of H_2_O_2_ across the plasma membrane, a steep gradient exists between extracellular and intracellular concentrations, with intracellular levels being approximately 390-fold (Lyublinskaya and Antunes, 2019) or even up to 650-fold (Sies, 2017) lower than those outside the cell. Accordingly, treatment with 5 μM H_2_O_2_ is expected to result in intracellular concentrations of approximately 7.7–12.5 nM, which remain within the upper physiological range. Therefore, the H_2_O_2_-mediated effects observed in our experiments are unlikely to reflect unphysiological oxidative stress.

To ensure that FGF2 treatment or withdrawal did not induce major shifts in hPSC energy metabolism that could confound comparisons between treated and untreated cells, we examined mitochondrial morphology. Regardless of FGF2 presence within the timeframes used in our experiments, mitochondria remained small, perinuclear, and exhibited undeveloped cristae—hallmarks of hPSCs (Folmes et al., 2011; Varum et al., 2011). As a positive control, we confirmed that treating cells with a high dose of the PDHK inhibitor DCA induced a dynamic remodeling of mitochondrial morphology, consistent with metabolic “maturation” and demonstrating the metabolic plasticity of our hPSC model. Importantly, the presence or absence of FGF2 did not alter this DCA-induced mitochondrial remodeling. Together, these results suggest that our comparisons between FGF2-treated and untreated cells are not confounded by underlying differences in metabolic phenotype.

Previous studies have demonstrated that PDH can translocate to nucleus to provide Ac-CoA for histone acetylation and importantly, that nuclear PDH is regulated differently than mitochondrial PDH (Nagaraj et al., 2017; Sutendra et al., 2014). It is therefore plausible that the FGF2–MEK1/2–ERK1/2–ROS axis may differentially regulate nuclear and mitochondrial PDH as well. In the present study we did not distinguish between these compartments, but verifying this distinction will be an interesting direction for future research.

While PDH catalyzes the production of acetyl-CoA, and our data demonstrate increased mitochondrial membrane potential, oxygen consumption, and altered levels of TCA cycle metabolites following FGF2 treatment—collectively suggesting enhanced TCA cycle activity and thus higher acetyl-CoA demand—we did not directly measure intracellular acetyl-CoA levels. This constitutes a limitation of our study. Instead, we infer changes in acetyl-CoA availability based on indirect metabolic and functional readouts. Our interpretation is supported by previous reports showing that hPSCs primarily rely on glycolysis- and, therefore, PDH-derived acetyl-CoA for histone acetylation (Jo et al., 2020; Moussaieff et al., 2015).

Another limitation of our study is the absence of data on tyrosine phosphorylation of PDHK1, which is known to enhance its activity (Hitosugi et al., 2011). Previous studies have shown ROS-mediated activation of PDHK1 (Cesi et al., 2017), which may result from ROS-induced inhibition of phosphatases responsible for PDHK1 dephosphorylation (Sommer et al., 2002; Wright et al., 2009). Such a mechanism could act synergistically with the proposed oxidative inactivation of PDP1, further contributing to the regulation of PDH activity under varying oxygen and redox conditions.

## 5 Conclusion

The role of FGF2 signaling in maintaining pluripotency in hPSCs has been extensively studied, with prior work focusing on its direct regulation of the core pluripotency transcription factors NANOG, OCT-4, and SOX2 (Dvorak et al., 2005; Haghighi et al., 2018; Kjartansdóttir et al., 2012; Yu et al., 2011). In this study, we expand on this understanding by demonstrating that FGF2 also enhances global histone acetylation, an established epigenetic hallmark of the pluripotent state (Jo et al., 2020).

We show that this effect is mediated through the MEK1/2–ERK1/2 pathway downstream of FGF2, which reduces intracellular ROS levels (Fojtík et al., 2021). This redox regulation leads to activation of pyruvate dehydrogenase (PDH), thereby increasing the production of Ac-CoA, a critical substrate for histone acetyltransferases. Importantly, this FGF2-mediated pathway affects not only chromatin state but also the expression of *NANOG*, and to a lesser extent, *SOX2* and *POU5F1*, linking metabolic activity directly to the transcriptional and epigenetic regulation of pluripotency.

Notably, these effects were observed exclusively under low oxygen conditions (5% O_2_). Mild hypoxia has been previously shown to support pluripotency and even reverse ongoing differentiation of hPSCs (Forristal et al., 2010; 2013; Mathieu et al., 2013; Varum et al., 2009; Yoshida et al., 2009). Our findings are therefore consistent with earlier reports describing the positive impact of low oxygen on the maintenance of the pluripotent state and provide a novel molecular mechanism contributing to this effect. This is physiologically relevant, as the blastocyst and its inner cell mass, the only *in vivo* source of pluripotent cells, naturally exists in the low oxygen environment of oviducts (Fischer and Bavister, 1993; Sciorio and Smith, 2019). Our data further support the notion that 5% O_2_ more closely mimics the native conditions of early embryonic development than the conventional 21% O_2_ often used *in vitro*.

**FIGURE 6:**
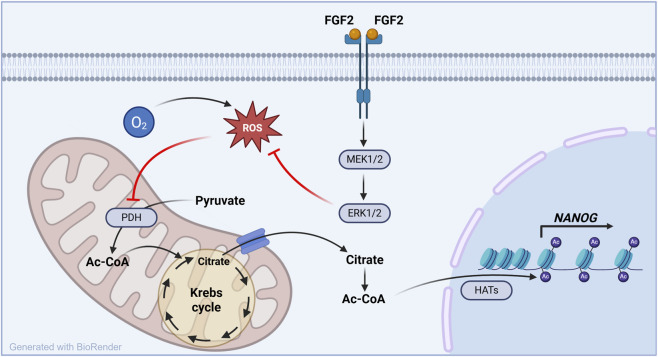
FGF2–MEK1/2–ERK1/2–ROS–PDH axis controls histone acetylation and NANOG expression under physiological oxygen conditions in hPSCs. This schematic model summarizes the mechanism uncovered in this study. Under low oxygen conditions (5% O_2_), FGF2 signaling reduces intracellular ROS levels through the MEK1/2–ERK1/2 pathway. This redox modulation activates pyruvate dehydrogenase (PDH), enhancing mitochondrial production of Ac-CoA, a key substrate for histone acetylation. The resulting increase in histone H3 acetylation promotes the expression of *NANOG*, a core pluripotency transcription factor. These effects are specific to 5% O_2_ and absent at 21% O_2_, highlighting the physiological relevance of oxygen tension in pluripotency regulation.

The ability to precisely control pluripotency is essential for the safe and effective use of hPSCs in basic research, disease modeling, and regenerative medicine. hPSCs are known to fluctuate between naïve and primed states (Hayashi et al., 2019; Kalmar et al., 2009; Nguyen et al., 2018), and both epigenetic marks and NANOG levels—the latter being highly dynamic—are central to this regulatory balance (Hayashi et al., 2019; Kalmar et al., 2009; M. Li and Izpisua Belmonte, 2018). Our study identifies the FGF2–MEK1/2-ERK1/2–ROS–PDH axis as a possible key modulator of these fluctuations, particularly under physiologically relevant oxygen conditions (Figure 6).

Taken together, our findings build on existing literature showing that glucose-derived Ac-CoA maintains histone acetylation in hPSCs (Jo et al., 2020; Moussaieff et al., 2015), and we further demonstrate that Ac-CoA production is dependent on PDH activity, which is in turn regulated by ROS homeostasis. This homeostasis is shaped by both oxygen availability and FGF2 signaling. Finally, we show that histone acetylation, driven by this pathway, supports expression of *NANOG*, thereby linking metabolic state to transcriptional control of pluripotency.

These insights can be used to optimize hPSC culture and differentiation protocols, particularly in contexts where ROS modulation plays a role, such as in cardiac and neuronal differentiation (Bell et al., 2015; Momtahan et al., 2019).

## Data Availability

The original contributions presented in the study are included in the article/Supplementary Material, further inquiries can be directed to the corresponding author.

## Glossary

2-OG: 2-oxoglutarate; alpha-ketoglutarate
Ac-CoA: Acetyl-CoA
AcH3: Acetylated histone H3
AKT: Protein kinase B
ACN: Acetonitrile
Cit: Sodium Citrate
CM+: Conditioned hESCM supplemented with FGF2
CM-: Conditioned hESCM without FGF2
CPI: CPI-613
esiRNA: Endoribonuclease-prepared small interfering RNA
FGF2: Fibroblast growth factor 2
FGFR: Fibroblast growth factor receptor
GSH: Glutathione (reduced)
**H**_**2**_ **O**_**2**_: Hydrogen peroxide
H3: Histone H3
HIF: Hypoxia-inducible factor
HIF-1a: Hypoxia-inducible factor 1 alpha
hESCM: Human embryonic stem cell media
hPSCs: Human pluripotent stem cells
LC-MS: Liquid chromatography coupled with mass spectrometry
mBCl: Monochlorobimane
MEK1/2: Mitogen activated protein kinase kinases 1/2
OxPhos: Oxidative phosphorylation
PCNA: Proliferating cell nuclear antigen
PD03: PD0325901
PDC: Pyruvate dehydrogenase complex
PDH: Pyruvate dehydrogenase
PDHK: Pyruvate dehydrogenase kinase
PDK1: Phosphoinositide-dependent kinase 1
PDP1: Pyruvate dehydrogenase phosphatase 1
PI3K: Phosphoinositide 3-kinase
ROS: Reactive oxygen species
Scr: Scrambled RNA control
TMRM: Tetramethylrhodamine methyl ester
Tu: Alpha tubulin
Vin: Vinculin

## References

[B1] Adewumi O. Aflatoonian B. Ahrlund-Richter L. Amit M. Andrews P. W. Beighton G. (2007). Characterization of human embryonic stem cell lines by the international stem cell initiative. Nat. Biotechnol. 25 (7), 803–816. 10.1038/nbt1318 17572666

[B2] Bell K. F. S. Al-Mubarak B. Martel M. A. McKay S. Wheelan N. Hasel P. (2015). Neuronal development is promoted by weakened intrinsic antioxidant defences due to epigenetic repression of Nrf2. Nat. Commun. 2015 6 (1), 7066–15. 10.1038/ncomms8066 25967870 PMC4441249

[B3] Carter B. Zhao K. (2020). The epigenetic basis of cellular heterogeneity. Nat. Rev. Genet. 22 (4), 235–250. 10.1038/s41576-020-00300-0 33244170 PMC10880028

[B4] Cerniglia G. J. Dey S. Gallagher-Colombo S. M. Daurio N. A. Tuttle S. Busch T. M. (2015). The PI3K/Akt pathway regulates oxygen metabolism *via* pyruvate dehydrogenase (PDH)-E1α phosphorylation. Mol. Cancer Ther. 14 (8), 1928–1938. 10.1158/1535-7163.MCT-14-0888 25995437 PMC4529780

[B5] Cesi G. Walbrecq G. Zimmer A. Kreis S. Haan C. (2017). ROS production induced by BRAF inhibitor treatment rewires metabolic processes affecting cell growth of melanoma cells. Mol. Cancer 16 (1), 1–16. 10.1186/S12943-017-0667-Y/FIGURES/9 28595656 PMC5465587

[B6] Chen G. Gulbranson D. R. Yu P. Hou Z. Thomson J. A. (2012). Thermal stability of fibroblast growth factor protein is a determinant factor in regulating self-renewal, differentiation, and reprogramming in human pluripotent stem cells. Stem Cells 30 (4), 623–630. 10.1002/stem.1021 22213113 PMC3538808

[B7] Cheung P. Tanner K. G. Cheung W. L. Sassone-Corsi P. Denu J. M. Allis C. D. (2000). Synergistic coupling of histone H3 phosphorylation and acetylation in response to epidermal growth factor stimulation. Mol. Cell 5 (6), 905–915. 10.1016/S1097-2765(00)80256-7 10911985

[B8] Chiarugi P. Cirri P. (2003). Redox regulation of protein tyrosine phosphatases during receptor tyrosine kinase signal transduction. Trends Biochem. Sci. 28 (9), 509–514. 10.1016/S0968-0004(03)00174-9 13678963

[B9] Choi H. W. Kim J. H. Chung M. K. Hong Y. J. Jang H. S. Seo B. J. (2015). Mitochondrial and metabolic remodeling during reprogramming and differentiation of the reprogrammed cells. Https//Home.Liebertpub.Com/Scd 24 (11), 1366–1373. 10.1089/SCD.2014.0561 25590788

[B10] Cui P. Zhang P. Zhang Y. Sun L. Cui G. Guo X. (2020). HIF-1α/Actl6a/H3K9ac axis is critical for pluripotency and lineage differentiation of human induced pluripotent stem cells. FASEB J. Official Publ. Fed. Am. Soc. Exp. Biol. 34 (4), 5740–5753. 10.1096/FJ.201902829RR 32112486

[B11] Dvorak P. Dvorakova D. Koskova S. Vodinska M. Najvirtova M. Krekac D. (2005). Expression and potential role of fibroblast growth factor 2 and its receptors in human embryonic stem cells. Stem Cells Dayt. Ohio 23 (8), 1200–1211. 10.1634/stemcells.2004-0303 15955829

[B12] Eiselleova L. Matulka K. Kriz V. Kunova M. Schmidtova Z. Neradil J. (2009). A complex role for FGF-2 in self-renewal, survival, and adhesion of human embryonic stem cells. Stem Cells 27 (8), 1847–1857. 10.1002/stem.128 19544431 PMC2798073

[B13] Fischer B. Bavister B. D. (1993). Oxygen tension in the oviduct and uterus of rhesus monkeys, hamsters and rabbits. J. Reproduction Fertil. 99 (2), 673–679. 10.1530/jrf.0.0990673 8107053

[B14] Fojtík P. Beckerová D. Holomková K. Šenfluk M. Rotrekl V. (2021). Both hypoxia-inducible factor 1 and MAPK signaling pathway attenuate PI3K/AKT *via* suppression of reactive oxygen species in human pluripotent stem cells. Front. Cell Dev. Biol. 8, 607444. 10.3389/fcell.2020.607444 33553145 PMC7859355

[B15] Folmes C. D. L. Nelson T. J. Martinez-Fernandez A. Arrell D. K. Lindor J. Z. Dzeja P. P. (2011). Somatic oxidative bioenergetics transitions into pluripotency-dependent glycolysis to facilitate nuclear reprogramming. Cell metab. 14 (2), 264–271. 10.1016/j.cmet.2011.06.011 21803296 PMC3156138

[B16] Forman H. J. Bernardo A. Davies K. J. A. (2016). What is the concentration of hydrogen peroxide in blood and plasma? Archives Biochem. Biophysics 603, 48–53. 10.1016/J.ABB.2016.05.005 27173735

[B17] Formenti F. Constantin-Teodosiu D. (2010). Regulation of human metabolism by hypoxia-inducible factor. Proc. The. 10.1073/pnas.1002339107/-/DCSupplemental PMC2906567 20616028

[B18] Forristal C. E. Wright K. L. Hanley N. A. Oreffo R. O. C. Houghton F. D. (2010). Hypoxia inducible factors regulate pluripotency and proliferation in human embryonic stem cells cultured at reduced oxygen tensions. Reprod. Camb. Engl. 139 (1), 85–97. 10.1530/REP-09-0300 19755485 PMC2791494

[B19] Forristal C. E. Christensen D. R. Chinnery F. E. Petruzzelli R. Parry K. L. Sanchez-Elsner T. (2013). Environmental oxygen tension regulates the energy metabolism and self-renewal of human embryonic stem cells. PLoS ONE 8 (5), 62507. 10.1371/journal.pone.0062507 23671606 PMC3645991

[B20] Fumarola C. Cretella D. La Monica S. Bonelli M. A. Alfieri R. Caffarra C. (2017). Enhancement of the anti-tumor activity of FGFR1 inhibition in squamous cell lung cancer by targeting downstream signaling involved in glucose metabolism. Oncotarget 8 (54), 91841–91859. 10.18632/ONCOTARGET.19279 29190880 PMC5696146

[B21] Geary L. Labonne C. (2018). FGF mediated mapk and pi3k/akt signals make distinct contributions to pluripotency and the establishment of neural crest. ELife 7, e33845. 10.7554/eLife.33845 29350613 PMC5790379

[B22] Goda N. Kanai M. (2012). Hypoxia-inducible factors and their roles in energy metabolism. Int. J. Hematol. 95 (5), 457–463. 10.1007/s12185-012-1069-y 22535382

[B23] Gottlob K. Majewski N. Kennedy S. Kandel E. Robey R. B. Hay N. (2001). Inhibition of early apoptotic events by Akt/PKB is dependent on the first committed step of glycolysis and mitochondrial hexokinase. Genes and Dev. 15 (11), 1406–1418. 10.1101/GAD.889901 11390360 PMC312709

[B24] Guo Y. Einhorn L. Kelley M. Hirota K. Yodoi J. Reinbold R. (2004). Redox regulation of the embryonic stem cell transcription factor Oct-4 by thioredoxin. STEM CELLS 22 (3), 259–264. 10.1634/STEMCELLS.22-3-259 15153603

[B25] Haghighi F. Dahlmann J. Nakhaei-Rad S. Lang A. Kutschka I. Zenker M. (2018). bFGF-mediated pluripotency maintenance in human induced pluripotent stem cells is associated with NRAS-MAPK signaling. Cell Commun. Signal. 16 (1), 96. 10.1186/s12964-018-0307-1 30518391 PMC6282345

[B26] Hattori N. Imao Y. Nishino K. Hattori N. Ohgane J. Yagi S. (2007). Epigenetic regulation of nanog gene in embryonic stem and trophoblast stem cells. Genes Cells 12 (3), 387–396. 10.1111/J.1365-2443.2007.01058.X 17352742

[B27] Hayashi Y. Ohnuma K. Furue M. K. (2019). Pluripotent stem cell heterogeneity. Adv. Exp. Med. Biol. 1123, 71–94. 10.1007/978-3-030-11096-3_6 31016596

[B28] Hedley D. W. Hallahan A. R. Tripp E. H. (1990). Flow cytometric measurement of glutathione content of human cancer biopsies. Br. J. Cancer 61 (1), 65–68. 10.1038/BJC.1990.14 2297492 PMC1971330

[B29] Hitosugi T. Fan J. Chung T. W. Lythgoe K. Wang X. Xie J. (2011). Tyrosine phosphorylation of mitochondrial pyruvate dehydrogenase kinase 1 is important for cancer metabolism. Mol. Cell 44 (6), 864–877. 10.1016/j.molcel.2011.10.015 22195962 PMC3246218

[B30] Horne G. A. Stewart H. J. S. Dickson J. Knapp S. Ramsahoye B. Chevassut T. (2014). Nanog requires BRD4 to maintain murine embryonic stem cell pluripotency and is suppressed by bromodomain inhibitor JQ1 together with Lefty1. Stem Cells Dev. 24 (7), 879–891. 10.1089/SCD.2014.0302 25393219 PMC4367495

[B31] Huang F. Abmayr S. M. Workman J. L. (2016). Regulation of KAT6 acetyltransferases and their roles in cell cycle progression, stem cell maintenance, and human disease. Regul. KAT6 Acetyltransferases Their Roles Cell Cycle Progression, Stem Cell Maintenance, Hum. Dis. 36, 1900–1907. 10.1128/MCB.00055-16 27185879 PMC4936061

[B32] Jin Q. Yu L. R. Wang L. Zhang Z. Kasper L. H. Lee J. E. (2011). Distinct roles of GCN5/PCAF-mediated H3K9ac and CBP/p300-mediated H3K18/27ac in nuclear receptor transactivation. EMBO J. 30 (2), 249–262. 10.1038/EMBOJ.2010.318 21131905 PMC3025463

[B33] Jo C. Park S. Oh S. Choi J. Kim E. K. Youn H. D. (2020). Histone acylation marks respond to metabolic perturbations and enable cellular adaptation. Exp. and Mol. Med. 52 (12), 2005–2019. 10.1038/s12276-020-00539-x 33311704 PMC8080766

[B34] Kalmar T. Lim C. Hayward P. Muñoz-Descalzo S. Nichols J. Garcia-Ojalvo J. (2009). Regulated fluctuations in nanog expression mediate cell fate decisions in embryonic stem cells. PLOS Biol. 7 (7), e1000149. 10.1371/JOURNAL.PBIO.1000149 19582141 PMC2700273

[B35] Karagiota A. Kanoura A. Paraskeva E. Simos G. Chachami G. (2022). Pyruvate dehydrogenase phosphatase 1 (PDP1) stimulates HIF activity by supporting histone acetylation under hypoxia. FEBS J. 290, 2165–2179. 10.1111/FEBS.16694 36453802

[B36] Kelly G. Grose R. Zhang J. Zhang J.-S. J-s Z. Mossahebi-Mohammadi M. (2020). FGF signaling pathway: a key regulator of stem cell pluripotency. Cell Dev. Biol. 8, 79. 10.3389/fcell.2020.00079 PMC7040165 32133359

[B37] Kim J. Tchernyshyov I. Semenza G. L. Dang C. V. (2006). HIF-1-mediated expression of pyruvate dehydrogenase kinase: a metabolic switch required for cellular adaptation to hypoxia. Cell Metab. 3 (3), 177–185. 10.1016/j.cmet.2006.02.002 16517405

[B38] Kim M. S. Cho H. I. Park S. H. Kim J. H. Chai Y. G. Jang Y. K. (2015). The histone acetyltransferase Myst2 regulates nanog expression, and is involved in maintaining pluripotency and self-renewal of embryonic stem cells. FEBS Lett. 589 (8), 941–950. 10.1016/J.FEBSLET.2015.02.029 25743411

[B39] Kim J. H. Choi T. G. Park S. Yun H. R. Nguyen N. N. Y. Jo Y. H. (2018). Mitochondrial ROS-Derived PTEN oxidation activates PI3K pathway for mTOR-induced myogenic autophagy. Cell Death Differ. 25 (11), 1921–1937. 10.1038/s41418-018-0165-9 30042494 PMC6219511

[B40] Kjartansdóttir K. R. Gabrielsen A. Reda A. Söder O. Bergström-Tengzelius R. Andersen C. Y. (2012). Differentiation of stem cells upon deprivation of exogenous FGF2: a general approach to study spontaneous differentiation of hESCs *in vitro* . Syst. Biol. Reproductive Med. 58 (6), 330–338. 10.3109/19396368.2012.694009 22708801 PMC3507279

[B41] Korotchkina L. G. Patel M. S. (2001). Site specificity of four pyruvate dehydrogenase kinase isoenzymes toward the three phosphorylation sites of human pyruvate dehydrogenase. J. Biol. Chem. 276 (40), 37223–37229. 10.1074/JBC.M103069200 11486000

[B42] Krutá M. Šeneklová M. Raška J. Salykin A. Zerzánková L. Pešl M. (2014). Mutation frequency dynamics in hprt locus in culture-adapted human embryonic stem cells and induced pluripotent stem cells correspond to their differentiated counterparts. Stem Cells Dev. 23 (20), 2443–2454. 10.1089/scd.2013.0611 24836366 PMC4186764

[B43] Kučera J. Netušilová J. Sladeček S. Lánová M. Vašíček O. Štefková K. (2017). Hypoxia downregulates MAPK/ERK but not STAT3 signaling in ROS-dependent and HIF-1-Independent manners in Mouse embryonic stem cells. *Oxidative Med. Cell. Longev. 2017*(ROS Oxidative Stress Stem Cells) 16, 4386947. 10.1155/2017/4386947 28819544 PMC5551543

[B44] Lees J. G. Cliff T. S. Gammilonghi A. Ryall J. G. Dalton S. Gardner D. K. (2019). Oxygen regulates human pluripotent stem cell metabolic flux. Stem Cells Int. 2019, 8195614. 10.1155/2019/8195614 31236115 PMC6545818

[B45] Levenson J. M. O’Riordan K. J. Brown K. D. Trinh M. A. Molfese D. L. Sweatt J. D. (2004). Regulation of histone acetylation during memory Formation in the hippocampus. J. Biol. Chem. 279 (39), 40545–40559. 10.1074/JBC.M402229200 15273246

[B46] Li M. Izpisua Belmonte J. C. (2018). Deconstructing the pluripotency gene regulatory network. Nat. Cell Biol. 20 (4), 382–392. 10.1038/s41556-018-0067-6 29593328 PMC6620196

[B47] Li J. Wang G. Wang C. Zhao Y. Zhang H. Tan Z. (2007). MEK/ERK signaling contributes to the maintenance of human embryonic stem cell self-renewal. Differentiation 75 (4), 299–307. 10.1111/j.1432-0436.2006.00143.x 17286604

[B48] Li Y. Gruber J. J. Litzenburger U. M. Zhou Y. Miao Y. R. LaGory E. L. (2020). Acetate supplementation restores chromatin accessibility and promotes tumor cell differentiation under hypoxia. Cell Death Dis. 11 (2), 102. 10.1038/S41419-020-2303-9 32029721 PMC7005271

[B49] Li A. M. He B. Karagiannis D. Li Y. Jiang H. Srinivasan P. (2023). Serine starvation silences estrogen receptor signaling through histone hypoacetylation. Proc. Natl. Acad. Sci. U. S. A. 120 (38), e2302489120. 10.1073/PNAS.2302489120 37695911 PMC10515173

[B50] Liemburg-Apers D. C. Willems P. H. G. M. Koopman W. J. H. Grefte S. (2015). Interactions between mitochondrial reactive oxygen species and cellular glucose metabolism. Archives Toxicol. 89 (8), 1209–1226. 10.1007/S00204-015-1520-Y 26047665 PMC4508370

[B51] Lin Q. Lee Y. J. Yun Z. (2006). Differentiation arrest by hypoxia. J. Biol. Chem. 281 (41), 30678–30683. 10.1074/jbc.C600120200 16926163

[B52] Liu J. Chen G. Liu Z. Liu S. Cai Z. You P. (2018). Aberrant FGFR tyrosine kinase signaling enhances the warburg effect by reprogramming LDH isoform expression and activity in prostate cancer. Cancer Res. 78 (16), 4459–4470. 10.1158/0008-5472.CAN-17-3226 29891507 PMC6095720

[B53] Lyublinskaya O. Antunes F. (2019). Measuring intracellular concentration of hydrogen peroxide with the use of genetically encoded H2O2 biosensor HyPer. Redox Biol. 24, 101200. 10.1016/J.REDOX.2019.101200 31030065 PMC6482347

[B54] Mathieu J. Zhang Z. Nelson A. Lamba D. A. Reh T. A. Ware C. (2013). Hypoxia induces re-entry of committed cells into pluripotency. Stem Cells 31 (9), 1737–1748. 10.1002/stem.1446 23765801 PMC3921075

[B55] Meng T. C. Fukada T. Tonks N. K. (2002). Reversible oxidation and inactivation of protein tyrosine phosphatases *in vivo* . Mol. Cell 9 (2), 387–399. 10.1016/S1097-2765(02)00445-8 11864611

[B56] Molavian H. R. Kohandel M. Sivaloganathan S. (2016). High concentrations of H2O2 make aerobic glycolysis energetically more favorable for cellular respiration. Front. Physiology 7 (AUG), 362. 10.3389/FPHYS.2016.00362 27601999 PMC4993762

[B57] Momtahan N. Crosby C. O. Zoldan J. (2019). The role of reactive oxygen species in *in vitro* cardiac maturation. Trends Mol. Med. 25 (6), 482–493. 10.1016/J.MOLMED.2019.04.005 31080142 PMC6915959

[B58] Moráň L. Pivetta T. Masuri S. Vašíčková K. Walter F. Prehn J. (2019). Mixed copper(II)–phenanthroline complexes induce cell death of ovarian cancer cells by evoking the unfolded protein response. Metallomics 11 (9), 1481–1489. 10.1039/C9MT00055K 31348483

[B59] Moussaieff A. Rouleau M. Kitsberg D. Cohen M. Levy G. Barasch D. (2015). Glycolysis-mediated changes in acetyl-CoA and histone acetylation control the early differentiation of embryonic stem cells. Cell Metab. 21 (3), 392–402. 10.1016/J.CMET.2015.02.002 25738455

[B60] Mu X. Yan S. Fu C. Wei A. (2015). The histone acetyltransferase MOF promotes induces generation of pluripotent stem cells. Https//Home.Liebertpub.Com/Cell 17 (4), 259–267. 10.1089/CELL.2014.0102 26091365

[B61] Mullarky E. Cantley L. C. (2015). Diverting glycolysis to combat oxidative stress. Innov. Med., 3–23. 10.1007/978-4-431-55651-0_1 29787184

[B62] Mylonis I. Chachami G. Paraskeva E. Simos G. (2008). Atypical CRM1-dependent nuclear export signal mediates regulation of hypoxia-inducible factor-1alpha by MAPK. J. Biol. Chem. 283 (41), 27620–27627. 10.1074/JBC.M803081200 18687685

[B63] Nagaraj R. Sharpley M. S. Chi F. Braas D. Zhou Y. Kim R. (2017). Nuclear localization of mitochondrial TCA cycle enzymes as a critical step in Mammalian zygotic genome activation. Cell 168 (1–2), 210–223.e11. 10.1016/j.cell.2016.12.026 28086092 PMC5321559

[B64] Napolitano G. Fasciolo G. Venditti P. (2021). Mitochondrial management of reactive oxygen species. Antioxidants 10 (11), 1824. 10.3390/ANTIOX10111824 34829696 PMC8614740

[B65] Närvä E. Pursiheimo J. P. Laiho A. Rahkonen N. Emani M. R. Viitala M. (2013). Continuous hypoxic culturing of human embryonic stem cells enhances SSEA-3 and MYC levels. PLoS ONE 8 (11), e78847. 10.1371/journal.pone.0078847 24236059 PMC3827269

[B66] Nguyen Q. H. Lukowski S. W. Chiu H. S. Senabouth A. Bruxner T. J. C. Christ A. N. (2018). Single-cell RNA-seq of human induced pluripotent stem cells reveals cellular heterogeneity and cell state transitions between subpopulations. Genome Res. 28 (7), 1053–1066. 10.1101/GR.223925.117 29752298 PMC6028138

[B67] Nyunoya T. Monick M. M. Powers L. S. Yarovinsky T. O. Hunninghake G. W. (2005). Macrophages survive hyperoxia *via* prolonged ERK activation due to phosphatase down-regulation. J. Biol. Chem. 280 (28), 26295–26302. 10.1074/jbc.M500185200 15901735

[B68] Okazaki K. Maltepe E. (2006). Oxygen, epigenetics and stem cell fate. Regen. Med. 1 (1), 71–83. 10.2217/17460751.1.1.71 17465821

[B69] Okoh V. O. Felty Q. Parkash J. Poppiti R. Roy D. (2013). Reactive oxygen species *via* Redox signaling to PI3K/AKT pathway contribute to the malignant growth of 4-Hydroxy estradiol-transformed mammary epithelial cells. PLoS ONE 8 (2), e54206. 10.1371/journal.pone.0054206 23437041 PMC3578838

[B70] Östman A. Frijhoff J. Sandin Å. Böhmer F. D. (2011). Regulation of protein tyrosine phosphatases by reversible oxidation. J. Biochem. 150 (4), 345–356. 10.1093/jb/mvr104 21856739

[B71] Pan G. Thomson J. A. (2007). Nanog and transcriptional networks in embryonic stem cell pluripotency. Cell Res. 17 (1), 42–49. 10.1038/SJ.CR.7310125 17211451

[B72] Papa S. Choy P. M. Bubici C. (2019). The ERK and JNK pathways in the regulation of metabolic reprogramming. Oncogene 38, 2223–2240. 10.1038/s41388-018-0582-8 30487597 PMC6398583

[B73] Papandreou I. Cairns R. a. Fontana L. Lim A. L. Denko N. C. (2006). HIF-1 mediates adaptation to hypoxia by actively downregulating mitochondrial oxygen consumption. Cell Metab. 3 (3), 187–197. 10.1016/j.cmet.2006.01.012 16517406

[B74] Patel M. S. Korotchkina L. G. (2006). Regulation of the pyruvate dehydrogenase complex. Biochem. Soc. Trans. 34 (Pt 2), 217–222. 10.1042/BST20060217 16545080

[B75] Petillo A. Abruzzese V. Koshal P. Ostuni A. Bisaccia F. (2020). Extracellular citrate is a trojan horse for cancer cells. Front. Mol. Biosci. 7, 593866. 10.3389/FMOLB.2020.593866 33282912 PMC7688668

[B76] Pieri L. Dominici S. Del Bello B. Maellaro E. Comporti M. Paolicchi A. (2003). Redox modulation of protein kinase/phosphatase balance in melanoma cells: the role of endogenous and γ-glutamyltransferase-dependent H2O2 production. Biochimica Biophysica Acta (BBA) - General Subj. 1621 (1), 76–83. 10.1016/S0304-4165(03)00048-5 12667613

[B77] Rardin M. J. Wiley S. E. Naviaux R. K. Murphy A. N. Jack E. (2009). Monitoring phosphorylation of the pyruvate dehydrogenase complex. Anal. Biochem. 389 (Issue 2), 157–164. 10.1016/j.ab.2009.03.040 19341700 PMC2713743

[B78] Richard D. E. Berra E. Gothié E. Roux D. Pouysségur J. (1999). p42/p44 mitogen-activated protein kinases phosphorylate hypoxia-inducible factor 1alpha (HIF-1alpha) and enhance the transcriptional activity of HIF-1. J. Biol. Chem. 274 (46), 32631–32637. 10.1074/JBC.274.46.32631 10551817

[B79] Robey R. B. Hay N. (2009). Is Akt the “Warburg kinase”?Akt-energy metabolism interactions and oncogenesis. Seminars Cancer Biol. 19 (1), 25–31. 10.1016/j.semcancer.2008.11.010 19130886 PMC2814453

[B80] Samanta D. Semenza G. L. (2017). Maintenance of redox homeostasis by hypoxia-inducible factors. Redox Biol. 13, 331–335. 10.1016/J.REDOX.2017.05.022 28624704 PMC5476461

[B81] Saxton R. A. Sabatini D. M. (2017). mTOR signaling in growth, metabolism, and disease. Cell 168 (6), 960–976. 10.1016/J.CELL.2017.02.004 28283069 PMC5394987

[B82] Sciorio R. Smith G. D. (2019). Embryo culture at a reduced oxygen concentration of 5%: a mini review. Zygote 27 (6), 355–361. 10.1017/S0967199419000522 31544720

[B83] Semenza G. L. (2001). HIF-1 and mechanisms of hypoxia sensing. Curr. Opin. Cell Biol. 13 (2), 167–171. 10.1016/s0955-0674(00)00194-0 11248550

[B84] Semenza G. L. (2011). Hypoxia-inducible factor 1: regulator of mitochondrial metabolism and mediator of ischemic preconditioning. Biochimica Biophysica Acta - Mol. Cell Res. 1813 (7), 1263–1268. 10.1016/j.bbamcr.2010.08.006 20732359 PMC3010308

[B85] Semenza G. L. Jiang B. H. Leung S. W. Passantino R. Concordat J. P. Maire P. (1996). Hypoxia response elements in the aldolase A, enolase 1, and lactate dehydrogenase a gene promoters contain essential binding sites for hypoxia-inducible factor 1. J. Biol. Chem. 271 (51), 32529–32537. 10.1074/jbc.271.51.32529 8955077

[B86] Shen Z. Wu Y. Manna A. Yi C. Cairns B. R. Evason K. J. (2024). Oct4 redox sensitivity potentiates reprogramming and differentiation. Genes Dev. 38 (7–8), 308–321. 10.1101/GAD.351411.123 38719541 PMC11146590

[B87] Sies H. (2017). Hydrogen peroxide as a central redox signaling molecule in physiological oxidative stress: oxidative eustress. Redox Biol. 11, 613–619. 10.1016/J.REDOX.2016.12.035 28110218 PMC5256672

[B88] Sies H. Jones D. P. (2020). Reactive oxygen species (ROS) as pleiotropic physiological signalling agents. Nat. Rev. Mol. Cell Biol. 2020 21 (7), 363–383. 10.1038/s41580-020-0230-3 32231263

[B89] Sinenko S. A. Starkova T. Y. Kuzmin A. A. Tomilin A. N. (2021). Physiological signaling functions of reactive oxygen species in stem cells: from flies to man. Front. Cell Dev. Biol. 9, 714370. 10.3389/fcell.2021.714370 34422833 PMC8377544

[B90] Smith E. R. Hewitson T. D. (2020). TGF-β1 is a regulator of the pyruvate dehydrogenase complex in fibroblasts. Sci. Rep. 10 (1), 17914. 10.1038/s41598-020-74919-8 33087819 PMC7578649

[B91] Sommer D. Coleman S. Swanson S. A. Stemmer P. M. (2002). Differential susceptibilities of serine/threonine phosphatases to oxidative and nitrosative stress. Archives Biochem. Biophysics 404 (2), 271–278. 10.1016/S0003-9861(02)00242-4 12147265

[B92] Sutendra G. Kinnaird A. Dromparis P. Paulin R. Stenson T. H. Haromy A. (2014). A nuclear pyruvate dehydrogenase complex is important for the generation of acetyl-CoA and histone acetylation. Cell 158 (1), 84–97. 10.1016/J.CELL.2014.04.046 24995980

[B93] Trisciuoglio D. Di Martile M. Del Bufalo D. (2018). Emerging role of histone acetyltransferase in stem cells and cancer. Stem Cells Int. 2018, 8908751. 10.1155/2018/8908751 30651738 PMC6311713

[B94] Tsogtbaatar E. Landin C. Minter-Dykhouse K. Folmes C. D. L. (2020). Energy metabolism regulates stem cell pluripotency. Front. Cell Dev. Biol. 8, 87. 10.3389/fcell.2020.00087 32181250 PMC7059177

[B95] Varum S. Momcilović O. Castro C. Ben-Yehudah a Ramalho-Santos J. Navara C. S. (2009). Enhancement of human embryonic stem cell pluripotency through inhibition of the mitochondrial respiratory chain. Stem Cell Res. 3 (2–3), 142–156. 10.1016/j.scr.2009.07.002 19716358 PMC2837416

[B96] Varum S. Rodrigues A. S. Moura M. B. Momcilovic O. Easley C. a. Ramalho-Santos J. (2011). Energy metabolism in human pluripotent stem cells and their differentiated counterparts. PloS One 6 (6), e20914. 10.1371/journal.pone.0020914 21698063 PMC3117868

[B97] Wang X. Q. Lo C. M. Chen L. Ngan E. S. W. Xu A. Poon R. Y. C. (2017). CDK1-PDK1-PI3K/Akt signaling pathway regulates embryonic and induced pluripotency. Cell Death Differ. 24 (1), 38–48. 10.1038/cdd.2016.84 27636107 PMC5260505

[B98] Wellen K. E. Hatzivassiliou G. Sachdeva U. M. Bui T. V. Cross J. R. Thompson C. B. (2009). ATP-citrate lyase links cellular metabolism to histone acetylation. Sci. (New York, N.Y.) 324 (5930), 1076–1080. 10.1126/SCIENCE.1164097 19461003 PMC2746744

[B99] Wright V. P. Reiser P. J. Clanton T. L. (2009). Redox modulation of global phosphatase activity and protein phosphorylation in intact skeletal muscle. J. Physiol. 587, 5767–5781. 10.1113/jphysiol.2009.178285 19841000 PMC2805384

[B100] Xie Y. Shi X. Sheng K. Han G. Li W. Zhao Q. (2019). PI3K/Akt signaling transduction pathway, erythropoiesis and glycolysis in hypoxia (Review). Mol. Med. Rep. 19 (2), 783–791. 10.3892/MMR.2018.9713 30535469 PMC6323245

[B101] Yang W. Zheng Y. Xia Y. Ji H. Chen X. Guo F. (2012). ERK1/2-dependent phosphorylation and nuclear translocation of PKM2 promotes the Warburg effect. Nat. Cell Biol. 14 (12), 1295–1304. 10.1038/NCB2629 23178880 PMC3511602

[B102] Yeaman S. J. Hutcheson E. T. Roch T. E. Pettit F. H. Brown J. R. Reed L. J. (1978). Sites of phosphorylation on pyruvate dehydrogenase from bovine kidney and heart. Biochemistry 17 (12), 2364–2370. 10.1021/BI00605A017 678513

[B103] Yoshida Y. Takahashi K. Okita K. Ichisaka T. Yamanaka S. (2009). Hypoxia enhances the generation of induced pluripotent stem cells. Cell Stem Cell 5 (3), 237–241. 10.1016/j.stem.2009.08.001 19716359

[B104] Yu P. Pan G. Yu J. Thomson J. A. (2011). FGF2 sustains NANOG and switches the outcome of BMP4-induced human embryonic stem cell differentiation. Cell Stem Cell 8 (3), 326–334. 10.1016/j.stem.2011.01.001 21362572 PMC3052735

[B105] Yucel N. Wang Y. X. Mai T. Porpiglia E. Lund P. J. Markov G. (2019). Glucose metabolism drives histone acetylation landscape transitions that dictate muscle stem cell function. Cell Rep. 27 (13), 3939–3955.e6. 10.1016/J.CELREP.2019.05.092 31242425 PMC6788807

[B106] Zhang J. Wang X. Vikash V. Ye Q. Wu D. Liu Y. (2016). ROS and ROS-Mediated cellular signaling. Oxidative Med. Cell. Longev. 2016, 4350965. 10.1155/2016/4350965 26998193 PMC4779832

[B107] Zhou D. Jiang L. Jin L. Yao Y. Wang P. Zhu X. (2020). Glucose transporter-1 cooperating with akt signaling promote gastric cancer progression. Cancer Manag. Res. 12, 4151–4160. 10.2147/CMAR.S251596 32581586 PMC7276340

