# Supplementary material for: FGF2 supports NANOG expression via pyruvate dehydrogenase–dependent histone acetylation under low oxygen conditions
Source: Front Cell Dev Biol. 2025 Oct 28;13:1623814. doi: 10.3389/fcell.2025.1623814 (PMC12602506; doi:10.3389/fcell.2025.1623814)
Supplement: Supplementary file 1 [file Presentation1.pdf]

List of Content

Supplementary Figures

- **Page 2: Figure S1**. 24-hour FGF2 starvation does not affect mitochondrial structure and development.
- **Page 3: Figure S2**. PDH inhibition leads to a decrease in histone acetylation, which can be rescued by acetyl-CoA precursors.
- **Page 4: Figure S3**. Activation of PDH using PDHKs inhibitor DCA does not increase H3 acetylation and NANOG levels.
- **Page 5: Figure S4**. Elevated ROS levels decrease acetylation of H3K9 and H3K27.
- **Page 6: Figure S5.** PDH phosphatase PDP1 is possibly responsible for ROS-dependent regulation of PDH phosphorylation.
- **Page 7: Figure S6.** Secondary data supplementing Figure 4.

Supplementary Tables

- **Pages 8-14: Table S7**. SRM transitions (Q1 → Q3, collision energy, and dwell time) of compounds analyzed in the intracellular content.

Figure S1. 24-hour FGF2 starvation does not affect mitochondrial structure and development.

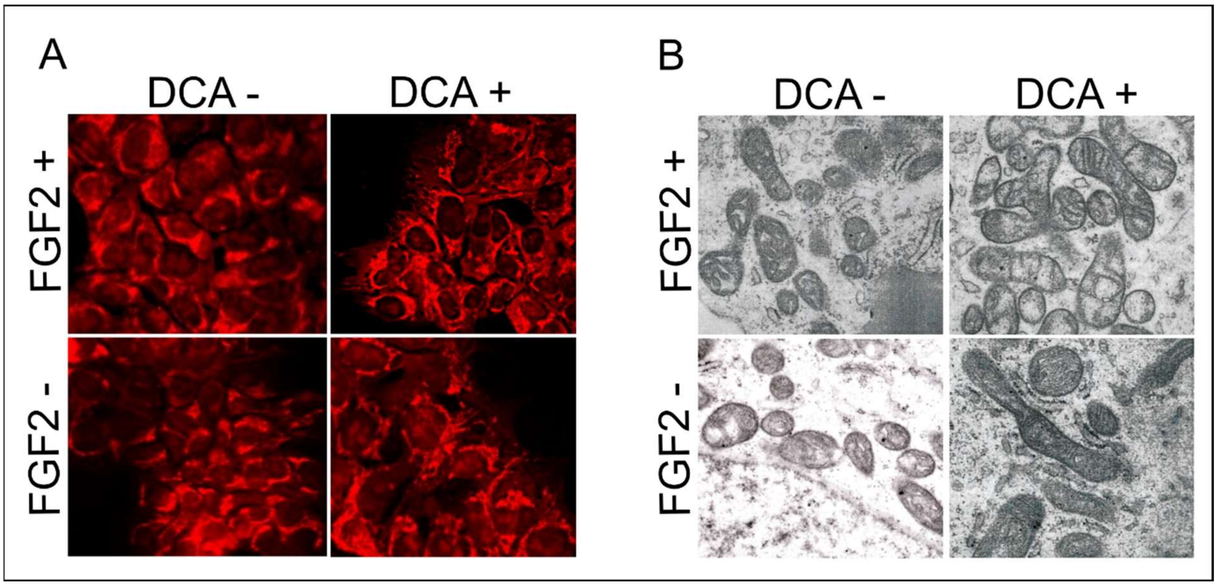

A) MitotrackerRed CMXRos staining of mitochondria in CCTL14 hPSCs.

B) Transmission electron microscopy of CCTL14 hPSCs with detail on the mitochondria

Figure S2. PDH inhibition leads to a decrease in histone acetylation, which can be rescued by acetyl-CoA precursors.

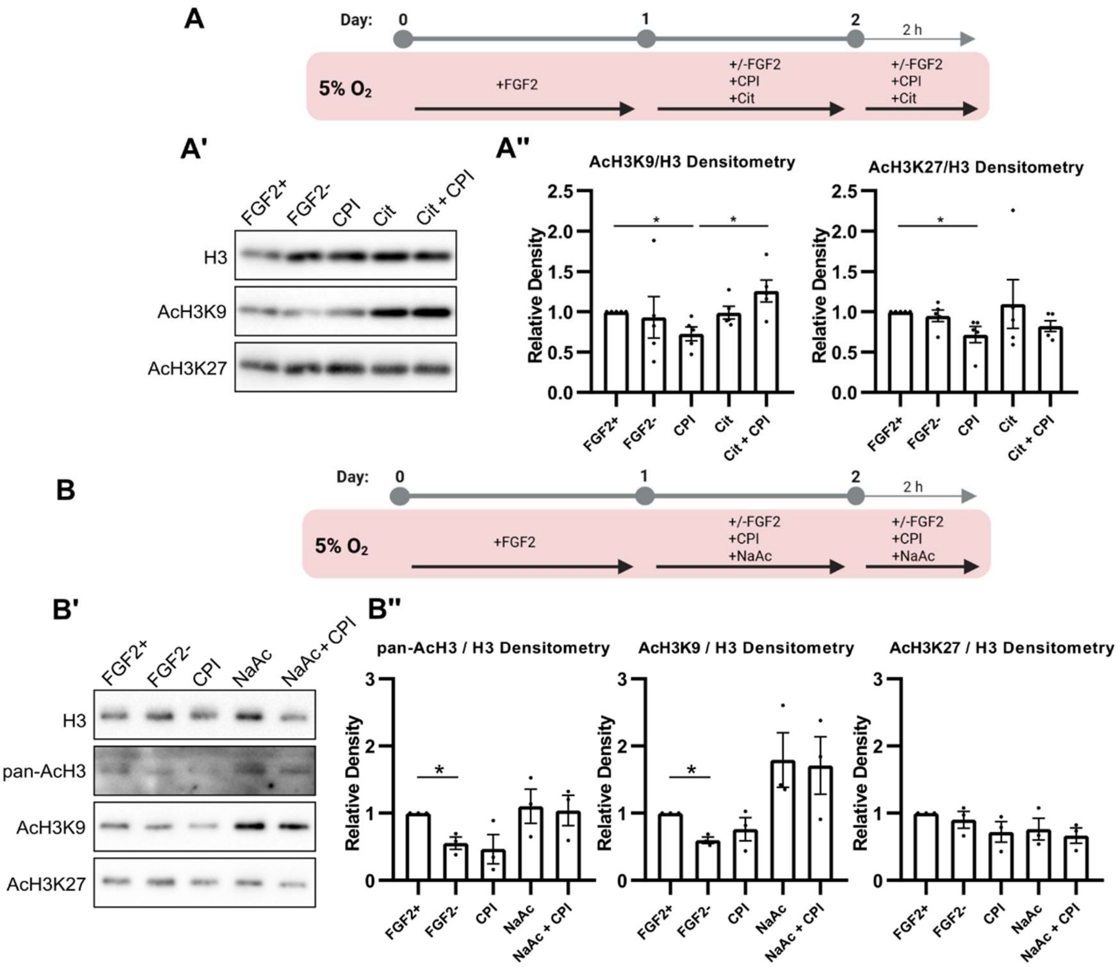

A) Schematic representation of experiment investigating the effect of PDH inhibition on H3 acetylation and rescue of this effect using sodium citrate.

A') Representative WB of PDH inhibition and its rescue in 5% O_2. Levels of total H3 and H3K9 and H3K27 acetylation were assayed. CPI decreased acetylation levels of both, and treatment with sodium citrate (Cit) rescued acetylation levels of H3K9.

A'') Densitometric analysis of 5 independent biological repetitions. Acetylated H3 was quantified as a ratio of acetylated to total H3.

B) Schematic representation of experiment investigating the effect of PDH inhibition on H3 acetylation and rescue of this effect using sodium acetate (NaAc).

B') Representative WB of PDH inhibition and its rescue in 5% O_2. Levels of total H3 and H3K9, H3K27, and H3 pan-acetylation were assayed. CPI decreased acetylation levels of H3 acetylation, and treatment with sodium acetate (NaAc) rescued H3 pan-acetylation and acetylation of H3K9.

B'') Densitometric analysis of 3 independent biological repetitions. Acetylated H3 was quantified as a ratio of acetylated to total H3.

Figure S3. Activation of PDH using PDHKs inhibitor DCA does not increase H3 acetylation and NANOG levels.

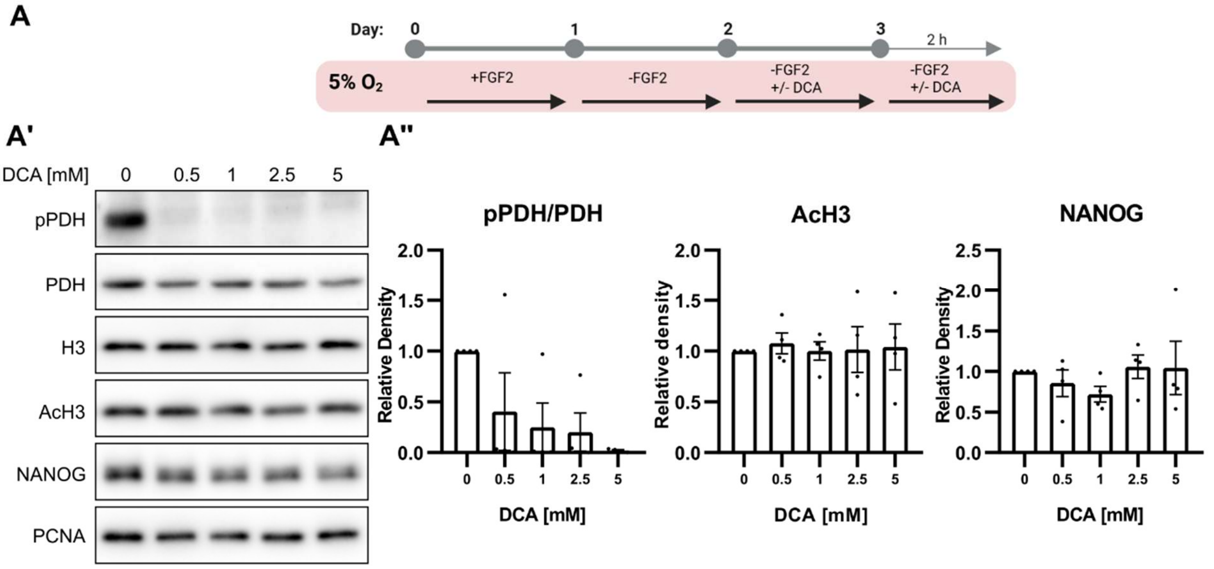

A) Schematic representation of experiment investigating the effect of PDH activation on AcH3 and NANOG levels in hESCs without FGF2.

A') Representative WB of DCA treatment of FGF2 deprived cells (FGF2-) in 5% O_2. hESCs were plated in 5% O2, deprived of FGF2 for 24 hours and then treated with various concentrations of DCA for 24 hours and another 2 hours on the next day. Levels of total and phosphorylated PDH, total and pan-acetylated histone H3, and NANOG were assayed. PCNA was used as a loading control.

A'') Densitometric analysis of 4 independent biological repetitions. DCA treatment increased the amount of active PDH (decreased PDH phosphorylation) in FGF2 deprived cells but had no significant changes on H3 pan-acetylation and NANOG levels were observed.

Figure S4. Elevated ROS levels decrease acetylation of H3K9 and H3K27.

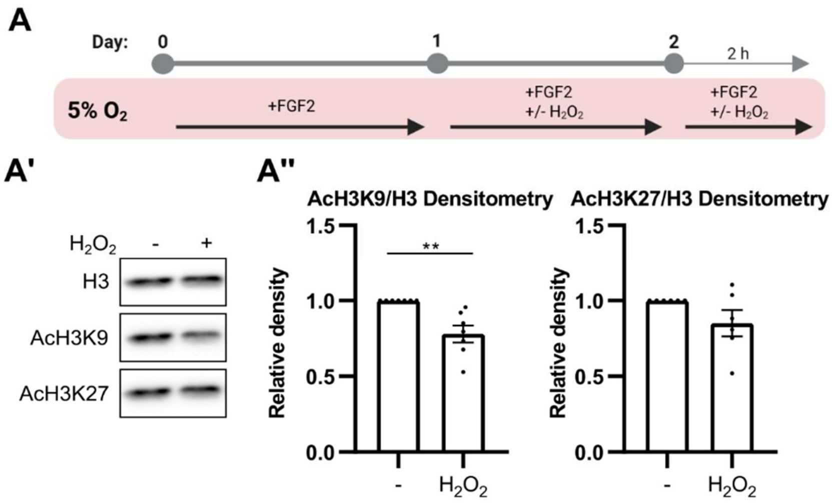

A) Schematic of the experimental workflow for analyzing the effect of ROS on H3 acetylation levels.

A') Representative Western blot of H3K9 and H3K27 acetylation following H_2O_2 treatment (5 μM, 24 + 2 h).

A'') Densitometric analysis of 6 independent biological repetitions. Acetylated H3 was quantified as a ratio of acetylated to total H3.

Figure S5. PDH phosphatase PDP1 is possibly responsible for ROS-dependent regulation of PDH phosphorylation.

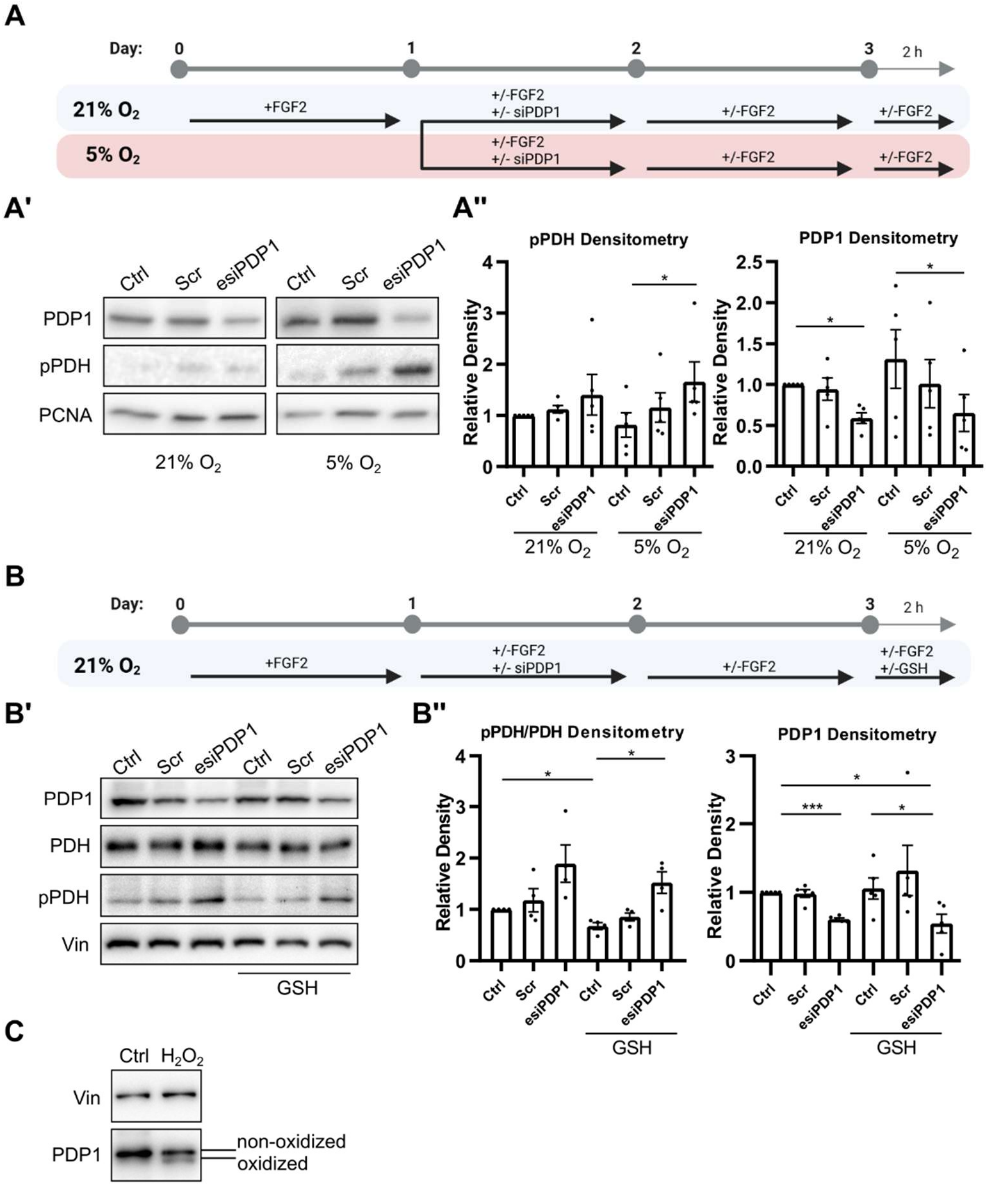

A) Schematic representation of experiment investigating the role of PDP1 in oxygen-dependent regulation of PDH activity.

A') Silencing of PDP1 expression increases PDH phosphorylation in 5% O_2. Cells transfected with scramble siRNA were used as a negative control. PCNA was used as a loading control.

A'') Densitometry of pPDH (N=5) and PDP1 (N=5) from CCTL14 hPSCs. Silencing of PDP1 leads to a significant increase in PDH phosphorylation in 5% O_2.

B) Schematic representation of experiment investigating the role of PDP1 in ROS-dependent regulation of PDH activity.

B') Silencing of PDP1 expression increases PDH phosphorylation in GSH-treated cells in 21% O_2. Cells transfected with scramble siRNA were used as a negative control. PCNA was used as a loading control.

B'') Densitometry of pPDH (N=5) and PDP1 (N=5) from CCTL14 and CCTL12 hPSCs. Cells treated with GSH show significant decrease in PDH phosphorylation. Cells with silenced PDP1 treated with GSH show significant increase in PDH phosphorylation.

C) WB analysis of PDP1 oxidation on CCTL14 hPSCs. Oxidized PDP1 migrated faster in the 8% native gel.

Figure S6. Secondary data supplementing Figure 4.

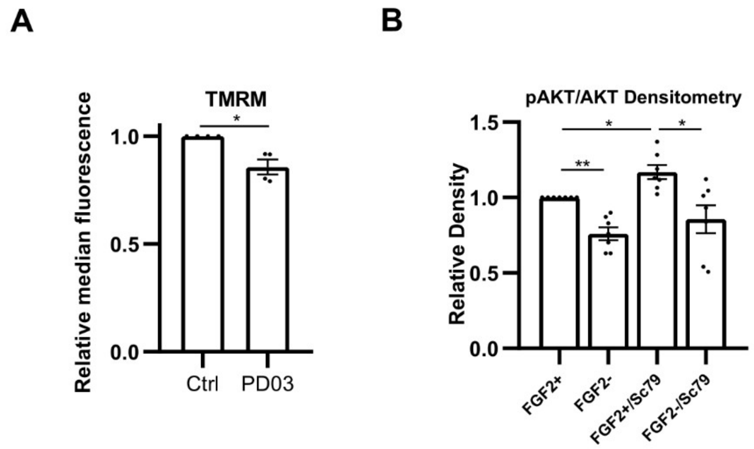

A) Measurement of mitochondrial membrane potential after MEK1/2 inhibition in 5% O_2 in CCTL14 hPSCs. Data represent the normalized median fluorescence ± SEM (N=4). Statistical analysis was performed using one sample t-test (theoretical mean = 1).

B) Densitometric analysis of AKT phosphorylation after treatment with AKT activator SC79 supplementing data in Figure 4C'. SC79 treatment significantly increased AKT phosphorylation.

Supplementary Tables

Table S7. SRM transitions (Q1 → Q3, collision energy, and dwell time) of compounds analyzed in the intracellular content.

Compound	Polarity	Q1 (m/z)	Q3 (m/z)	CE (eV)	Dwell time (ms)
2-OG	–	145.0	101.1	40	10.0
	–	145.0	57.3	7	10.0
3-Phosphoglycerate	–	185.0	97.1	9	7.5
	–	185.0	79.1	24	7.5
	–	185.0	167.0	3	7.5
6-Phosphogluconate	–	275.1	97.1	10	12.5
	–	275.1	79.1	26	12.5
	–	275.1	177.1	10	12.5
	–	275.1	99.1	18	12.5
8-hydroxy deoxyguanosin	+	284.0	168.1	-11	11.3
	+	284.0	117.2	-12	11.3
Acetyl-CoA	+	810.0	302.8	-26	5.0
	+	810.0	427.9	-24	5.0
	+	810.0	201.0	-29	5.0
	+	810.0	135.9	-47	5.0
Adenosine	+	268.1	136.1	-13	10.0
	+	268.1	119.1	-44	10.0
ADP	+	428.0	136.1	-15	20.0
Ala	+	90.1	44.1	-9	11.3
	+	90.1	43.2	-9	11.3
AMP	–	346.0	210.9	10	16.7
	–	346.0	79.2	25	16.7
	–	346.0	134.1	23	16.7
Arg	+	175.2	70.1	-25	11.3
	+	175.2	60.1	-25	11.3

Ascorbic acid	–	175.0	87.2	14	6.7
	–	175.0	115.1	10	6.7
	–	175.0	71.2	7	6.7
Asn	+	133.1	74.2	-12	7.5
	+	133.1	87.2	-7	7.5
	+	133.1	116.1	-7	7.5
Asp	–	132.0	88.2	7	11.3
	–	132.0	115.1	7	11.3
ATP	–	506.0	159.0	20	16.7
	–	506.0	115.0	20	16.7
	–	506.0	408.0	20	16.7
	+	508.0	136.1	-20	22.5
B12	+	678.6	359.0	-25	11.3
	+	678.6	456.6	-30	11.3
cAMP	–	328.1	134.1	20	16.7
	–	328.1	79.2	26	16.7
	–	328.1	107.2	40	16.7
	+	330.2	136.1	-22	16.7
	+	330.2	119.1	-44	16.7
	+	330.2	312.0	-12	16.7
Citric acid	–	191.0	111.1	9	7.5
	–	191.0	87.2	17	7.5
	–	191.0	85.2	11	7.5
Citrulline	+	176.1	159.1	-6	6.7
	+	176.1	70.3	-18	6.7
	+	176.1	113.2	-12	6.7
CMP	+	324.0	112.1	-10	7.5
	+	324.0	279.1	-10	7.5

	+	324.0	307.1	-5	7.5
	–	322.0	210.9	10	7.5
	–	322.0	79.1	24	7.5
	–	322.0	97.1	21	7.5
Creatinine	+	114.1	44.4	-15	10.0
	+	114.1	86.2	-8	10.0
Cys	+	122.0	59.3	-19	7.5
	+	122.0	76.2	-10	7.5
	+	122.0	105.1	-7	7.5
Folic acid	+	442.1	295.0	-22	16.7
	+	442.1	176.0	-38	16.7
	+	442.1	120.1	-32	16.7
Fru-1,6-BisP	–	339.0	97.1	14	16.7
	–	339.0	79.1	29	16.7
	–	339.0	240.9	7	16.7
Fumarate	–	115.0	71.2	4	20.0
Glu	+	148.1	84.2	-13	7.5
	+	148.1	130.1	-6	7.5
	+	148.1	102.2	-9	7.5
Glucuronic acid	–	193.0	113.1	6	16.7
	–	193.0	59.3	13	16.7
	–	193.0	73.2	10	16.7
Gly	+	76.0	30.5	-7	10.0
	+	76.0	48.4	-4	10.0
GMP	+	364.0	152.1	-11	6.7
	+	364.0	135.0	-35	6.7
	+	364.0	110.2	-42	6.7
GSH	+	308.1	76.2	-15	7.5

	+	308.1	84.2	-15	7.5
	+	308.1	161.9	-15	7.5
Guanosine	+	284.1	152.1	-10	7.5
	+	284.1	135.1	-36	7.5
	+	284.1	110.1	-37	7.5
Hex-6-P	–	259.0	97.1	10	11.3
	–	259.0	79.1	29	11.3
Hexose	–	179.1	59.3	15	7.5
	–	179.1	89.2	4	7.5
	–	179.1	119.1	4	7.5
His	+	156.2	110.0	-13	11.3
	+	156.2	83.1	-13	11.3
Homocysteine	+	136.0	90.2	-11	10.0
	+	136.0	118.1	-3	10.0
Ile	+	132.1	86.1	-15	11.3
	+	132.1	69.1	-15	11.3
Lactate	–	89.0	43.4	6	10.0
	–	89.0	45.3	9	10.0
Leu	+	132.1	86.1	-15	11.3
	+	132.1	69.1	-15	11.3
Lys	+	147.2	84.1	-17	22.5
Malate	–	133.0	115.0	4	6.7
	–	133.0	71.2	13	6.7
	–	133.0	73.2	16	6.7
Met	+	150.0	56.3	-14	11.3
	+	150.0	104.2	-9	11.3
Methylcobalamine	+	672.8	665.0	-5	6.7
	+	672.8	147.1	-43	6.7

	+	672.8	359.0	-26	6.7
Methyl-tetrahydrofolate	+	460.2	313.1	-18	16.7
	+	460.2	180.0	-36	16.7
	+	460.2	194.1	-32	16.7
NAD+	+	664.1	428.0	-25	11.3
	+	664.1	348.1	-35	11.3
NADH	+	666.1	514.1	-25	7.5
	+	666.1	428.0	-15	7.5
	+	666.1	302.0	-20	7.5
NADP+	+	744.1	136.2	-50	12.5
	+	744.1	507.9	-26	12.5
	+	744.1	604.1	-16	12.5
	+	744.1	622.1	-8	12.5
	+	372.0	123.1	-8	11.3
	+	372.0	136.1	-21	11.3
NADPH	–	744.1	159.0	47	12.5
	–	744.1	79.1	53	12.5
	–	744.1	621.9	8	12.5
	–	744.1	396.8	32	12.5
	–	371.5	79.1	24	12.5
	–	371.5	134.1	13	12.5
	–	371.5	304.0	5	12.5
	–	371.5	158.9	20	12.5
OH-Pro	+	132.0	86.2	-10	7.5
	+	132.0	68.3	-16	7.5
	+	132.0	41.4	-29	7.5
Orn	+	133.2	70.1	-17	11.3
	+	133.2	116.0	-17	11.3

Panthotenic acid	+	220.1	98.0	-25	11.3
	+	220.1	124.1	-22	11.3
PEP	–	167.0	79.1	6	20.0
Phe	+	166.2	120.0	-13	11.3
	+	166.2	77.1	-13	11.3
Pro	+	116.1	70.1	-17	11.3
	+	116.1	43.2	-17	11.3
Pyridoxine	+	170.1	134.1	-25	11.3
	+	170.1	106.1	-26	11.3
Ribose-5-P	–	229.0	97.1	8	11.3
	–	229.0	79.1	24	11.3
SAH	+	385.1	136.1	-19	11.3
	+	385.1	134.1	-19	11.3
SAM	+	399.1	250.1	-12	7.5
	+	399.1	136.1	-19	7.5
	+	399.1	298.1	-11	7.5
Ser	+	106.0	60.3	-9	7.5
	+	106.0	42.4	-19	7.5
	+	106.0	88.2	-6	7.5
Succinate	–	117.0	73.2	8	10.0
	–	117.0	99.1	6	10.0
Sulphate	–	97.1	80.1	21	20.0
Taurine	–	124.0	80.1	17	20.0
Tetrahydrofolate	+	446.2	166.1	-11	16.7
	+	446.2	299.1	-16	16.7
	+	446.2	178.1	-11	16.7
Thr	+	120.1	74.3	-8	11.3
	+	120.1	56.3	-13	11.3

Trp	+	205.1	188.0	-5	11.3
	+	205.1	146.0	-5	11.3
Tyr	+	182.2	136.0	-9	11.3
	+	182.2	165.0	-9	11.3
Uridine	+	245.1	113.1	-6	11.3
	+	245.1	70.2	-24	11.3
Val	+	118.1	72.3	-8	11.3
	+	118.1	55.4	-17	11.3
