# Supplementary figures and images for: FGF2 supports NANOG expression via pyruvate dehydrogenase–dependent histone acetylation under low oxygen conditions
Source: Front Cell Dev Biol. 2025 Oct 28;13:1623814. doi: 10.3389/fcell.2025.1623814 (PMC12602506; doi:10.3389/fcell.2025.1623814)

Figure 1 – source WB data

B

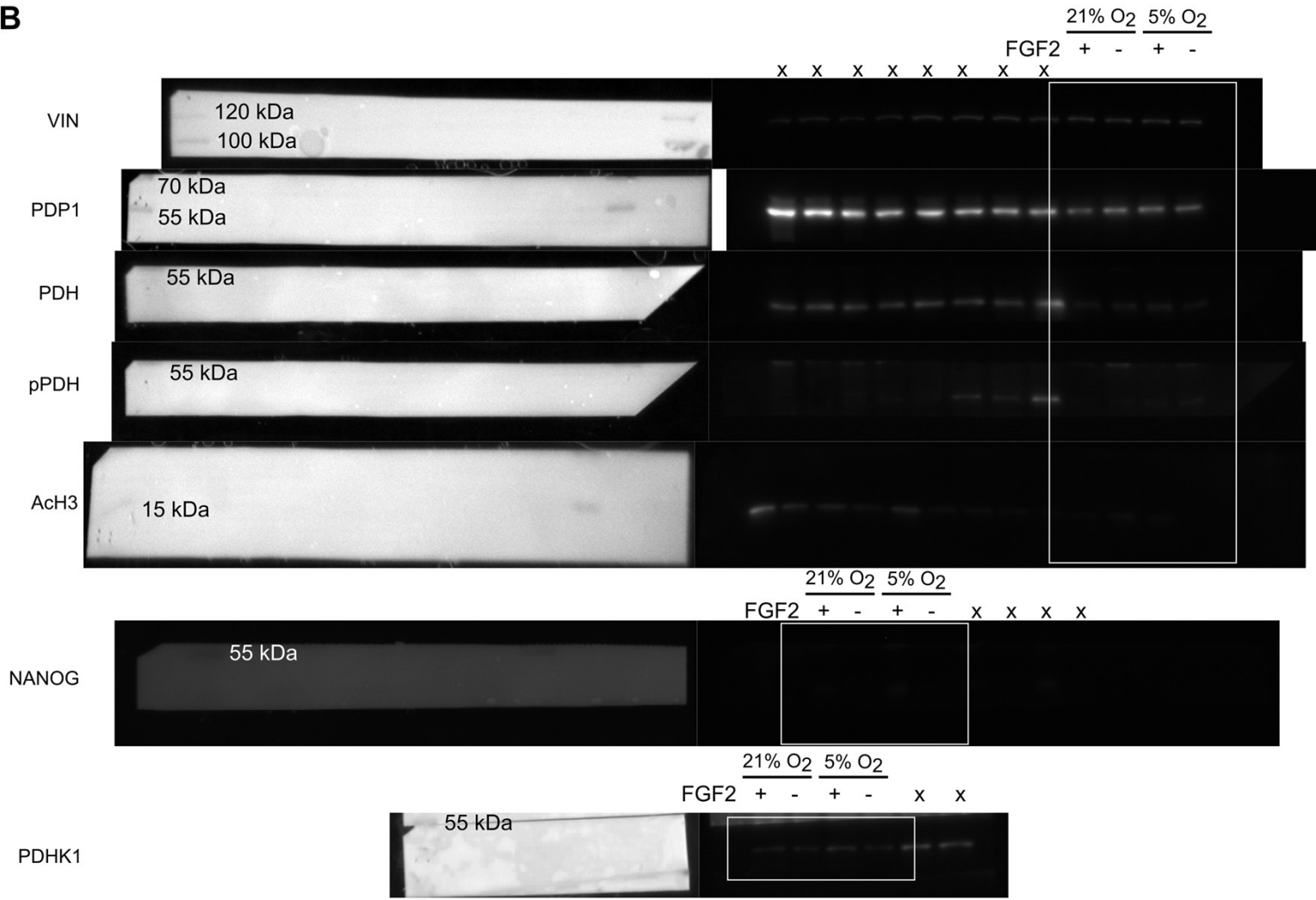

Figure 2 – source WB data

c

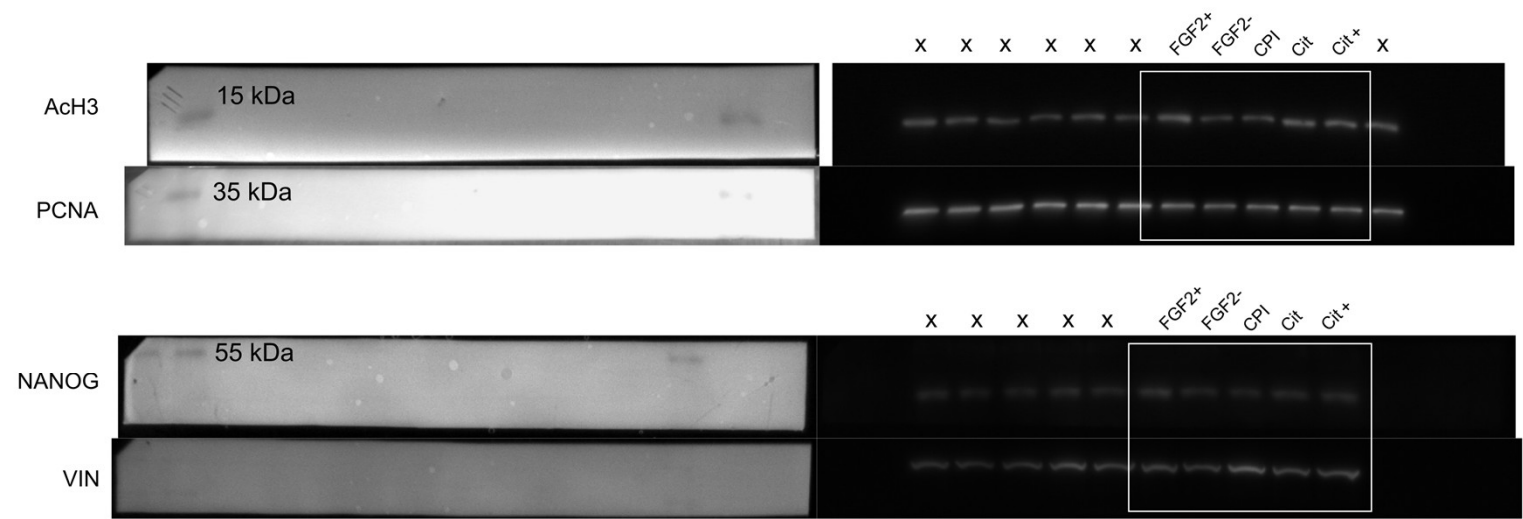

Figure 3 – source WB data

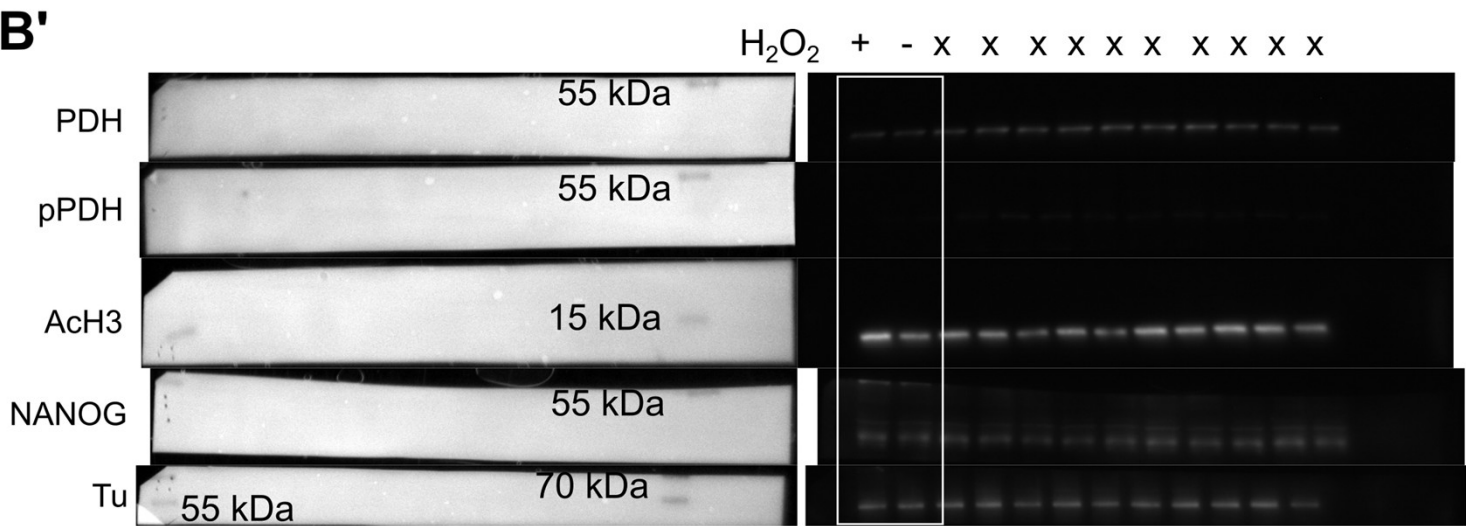

**A'**

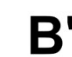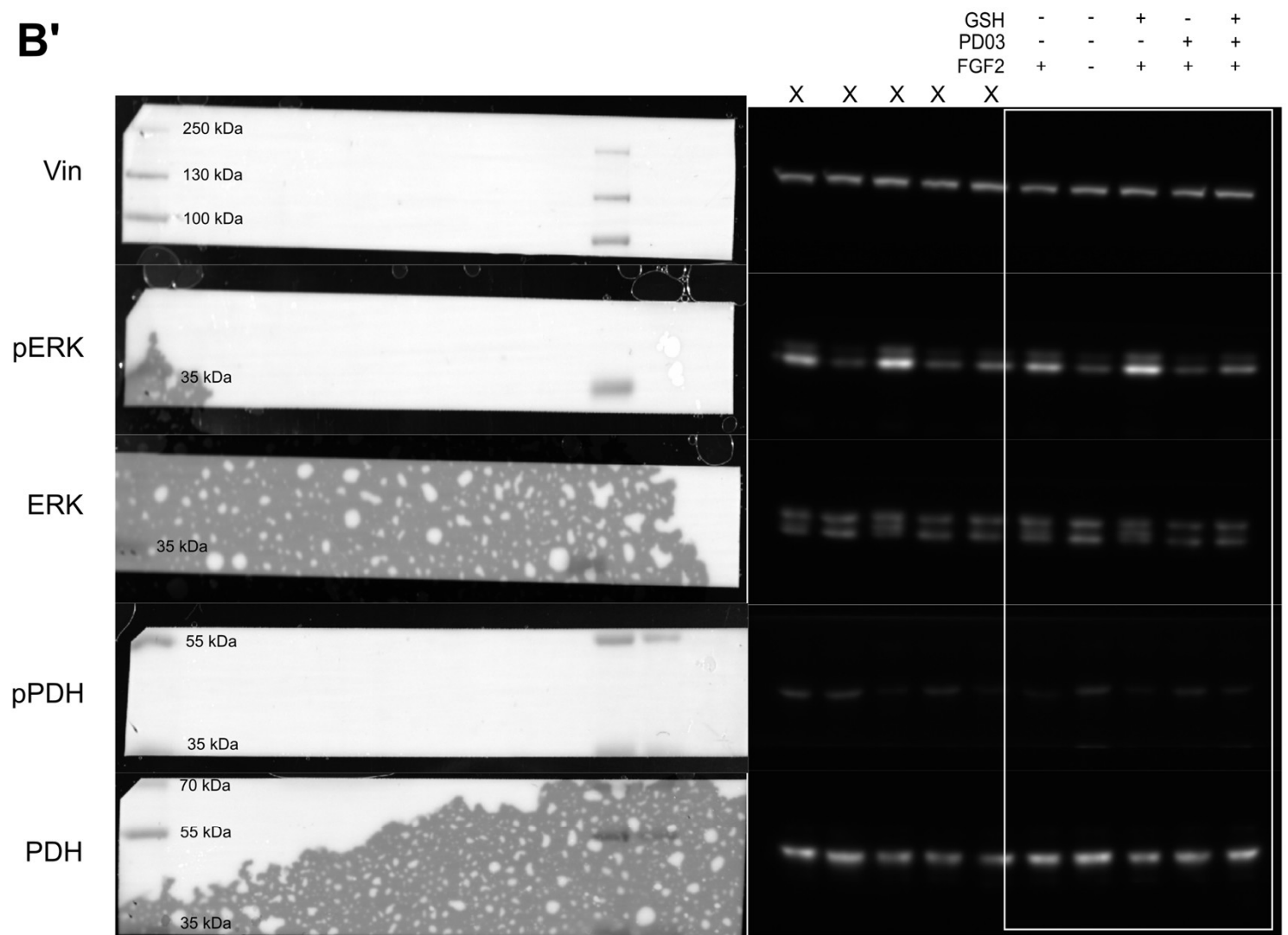

Figure 4 – source WB data

C'

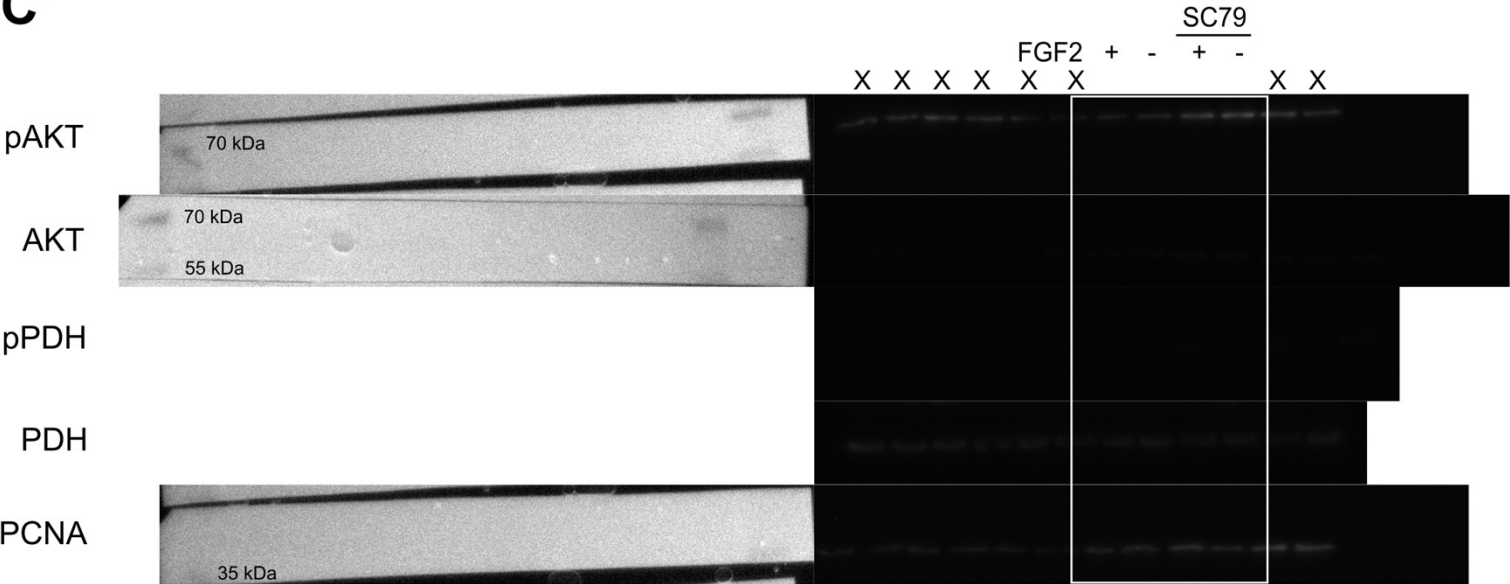

Supplement: Supplementary file 2 [file Presentation2.pdf]
